# TSPAN5 Enriched Microdomains Provide a Platform for Dendritic Spine Maturation through Neuroligin-1 Clustering

**DOI:** 10.1016/j.celrep.2019.09.051

**Published:** 2019-10-30

**Authors:** Edoardo Moretto, Anna Longatti, Luca Murru, Ingrid Chamma, Alessandro Sessa, Jonathan Zapata, Eric Hosy, Matthieu Sainlos, Julien Saint-Pol, Eric Rubinstein, Daniel Choquet, Vania Broccoli, Giampietro Schiavo, Olivier Thoumine, Maria Passafaro

**Affiliations:** 1 CNR, Institute of Neuroscience, Milan 20129, Italy; 2 BioMETRA, Department of Medical Biotechnology and Translational Medicine, University of Milan, Milan, Italy; 3 UK Dementia Research Institute at University College London, London WC1E 6BT, UK; 4 DiSFeB, Department of Pharmacological and Biomolecular Sciences, University of Milan, Milan, Italy; 5 Interdisciplinary Institute for Neuroscience, UMR 5297, Centre National de la Recherche Scientifique, Bordeaux, France; 6 Interdisciplinary Institute for Neuroscience, University of Bordeaux, Bordeaux, France; 7 Stem Cell and Neurogenesis Unit, Division of Neuroscience, San Raffaele Scientific Institute, 20132 Milan, Italy; 8 INSERM, U935, 94807 Villejuif, France; 9 Bordeaux Imaging Center, UMS3420, CNRS, University of Bordeaux, US4 INSERM, Bordeaux, France; 10 Department of Neuromuscular Diseases, UCL Queen Square Institute of Neurology, University College London, London WC1N 3BG, UK; 11 Discoveries Centre for Regenerative and Precision Medicine, University College London Campus, London WC1N 3BG, UK

**Keywords:** tetraspanin, TSPAN5, TEMs, dendritic spines, synapses, neuroligin-1, clustering

## Abstract

Tetraspanins are a class of evolutionarily conserved transmembrane proteins with 33 members identified in mammals that have the ability to organize specific membrane domains, named tetraspanin-enriched microdomains (TEMs). Despite the relative abundance of different tetraspanins in the CNS, few studies have explored their role at synapses. Here, we investigate the function of TSPAN5, a member of the tetraspanin superfamily for which mRNA transcripts are found at high levels in the mouse brain. We demonstrate that TSPAN5 is localized in dendritic spines of pyramidal excitatory neurons and that TSPAN5 knockdown induces a dramatic decrease in spine number because of defects in the spine maturation process. Moreover, we show that TSPAN5 interacts with the postsynaptic adhesion molecule neuroligin-1, promoting its correct surface clustering. We propose that membrane compartmentalization by tetraspanins represents an additional mechanism for regulating excitatory synapses.

## Highlights

• TSPAN5 is expressed in pyramidal neurons and localizes mainly to dendritic spines
• TSPAN5 interacts with neuroligin-1 and promotes its clustering
• TSPAN5-neuroligin-1 complex is fundamental for dendritic spine maturation

## Graphical Abstract

**Figure:**
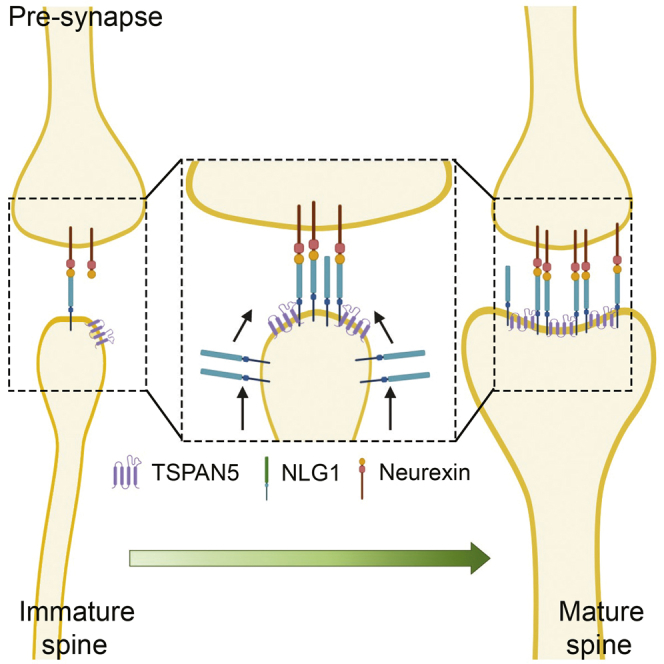

## Teaser

Moretto et al. demonstrate that TSPAN5 controls the maturation of dendritic spines by promoting the clustering of neuroligin-1. These findings provide proof of principle that compartmentalization of transmembrane proteins through tetraspanins can represent an additional level of regulation of synapse formation and function.

## Introduction

Tetraspanins are a class of transmembrane proteins evolutionarily conserved in metazoans. They share a common structure: four transmembrane domains (TMs), a small extracellular loop (SEL) and a large extracellular loop (LEL), and intracellular N and C termini (Berditchevski, 2001). Tetraspanins self-organize by homophilic interactions and accumulate in specialized membrane domains called tetraspanin-enriched microdomains (TEMs); this enables the concentration of other transmembrane proteins in segregated membrane domains (Charrin et al., 2002, Charrin et al., 2009, Charrin et al., 2003b, Charrin et al., 2014, Maecker et al., 1997, Boucheix and Rubinstein, 2001, van Deventer et al., 2017). Tetraspanins also bind cholesterol, an interaction thought to mediate their self-association (Charrin et al., 2003a). To date, 33 mammalian tetraspanins have been described to act in cell-cell adhesion, cell motility and proliferation, immunity, and nervous system development (Hemler, 2005), but there is little work on their synaptic function (Bassani et al., 2012, Murru et al., 2017, Murru et al., 2018), thus their supramolecular organization at synapses is unknown.

The role of these proteins in membrane domain assembly and in cell-cell adhesion supports a possible function in synapse formation and activity.

TSPAN5 belongs to the C8 subgroup of tetraspanins, characterized by eight cysteine residues forming disulfide bridges key for proper protein folding. Structure-function studies found TSPAN5 to act in cell fusion during osteoclastogenesis (Iwai et al., 2007, Zhou et al., 2014) and regulation of intracellular trafficking and function of ADAM-10 (Dornier et al., 2012, Haining et al., 2012, Jouannet et al., 2016, Noy et al., 2016, Saint-Pol et al., 2017).

Previous work analyzing spatial and temporal localization of *Tspan5* mRNA in mice revealed highest expression in the brain (García-Frigola et al., 2000), mostly in the hippocampus, neocortex, and Purkinje cells of the cerebellum, suggesting a neuronal enrichment (García-Frigola et al., 2001). *Tspan5* expression rises during postnatal development in all brain (García-Frigola et al., 2001, Juenger et al., 2005).

Here, we report that TSPAN5 is enriched in dendritic spines of rodent pyramidal neurons and that TSPAN5 modulation affects the maturation of dendritic spine by regulating neuroligin-1 (NLG1) clustering without strongly affecting excitatory synapse density or functionality.

Dendritic spines, small dendritic protrusions where the postsynaptic compartment of excitatory synapses is formed, are typically sorted by morphology as thin, stubby, and mushroom. Their maturation process is complex and still debated, but “mushroom” spines are considered the final mature morphological subtype (Yuste and Bonhoeffer, 2004).

NLG1, a major adhesion molecule of excitatory synapses (Bemben et al., 2015, Südhof, 2008), has roles in postsynaptic compartment assembly, synapses formation and strengthening by binding to presynaptic neurexins (Bemben et al., 2015). However, the exact contribution of NLG1 during dendritic spine and excitatory synapse formation is still debated (Chanda et al., 2017, Kwon et al., 2012, Letellier et al., 2018).

Our data reveal TSPAN5 as a regulator of synapse organization and support the role for membrane compartmentalization of NLG1 in this process.

## Results

### TSPAN5 Is Expressed in the Postsynaptic Compartment of Hippocampal Pyramidal Neurons

TSPAN5 has not been studied in neurons; thus, we confirmed its expression in mouse brain lysates obtained with strong detergents (Triton X-100 and NP40) (Figure S1A, WT lane).

We validated the specificity of the α-TSPAN5 antibody on brain lysates from TSPAN5-knockout mice (Saint-Pol et al., 2017) (Figure S1A) and on extracts from HeLa cells transfected with either TSPAN5-GFP or other tetraspanins such as CD9-GFP and CD81-GFP (Figure S1B).

We confirmed TSPAN5 localization at the neuronal plasma membrane in cultured hippocampal neurons (Figure S1C) and adult mice brain slices (Figure S1D) using the membrane impermeable crosslinker bis(sulfosuccinimidyl)suberate (BS3). Bands detected by the α-TSPAN5 antibody are specific, as confirmed by the reduction of band intensity by a previously published short hairpin RNA (shRNA) for TSPAN5 (Figure S1F) (Dunn et al., 2010).

**Figure 1:**
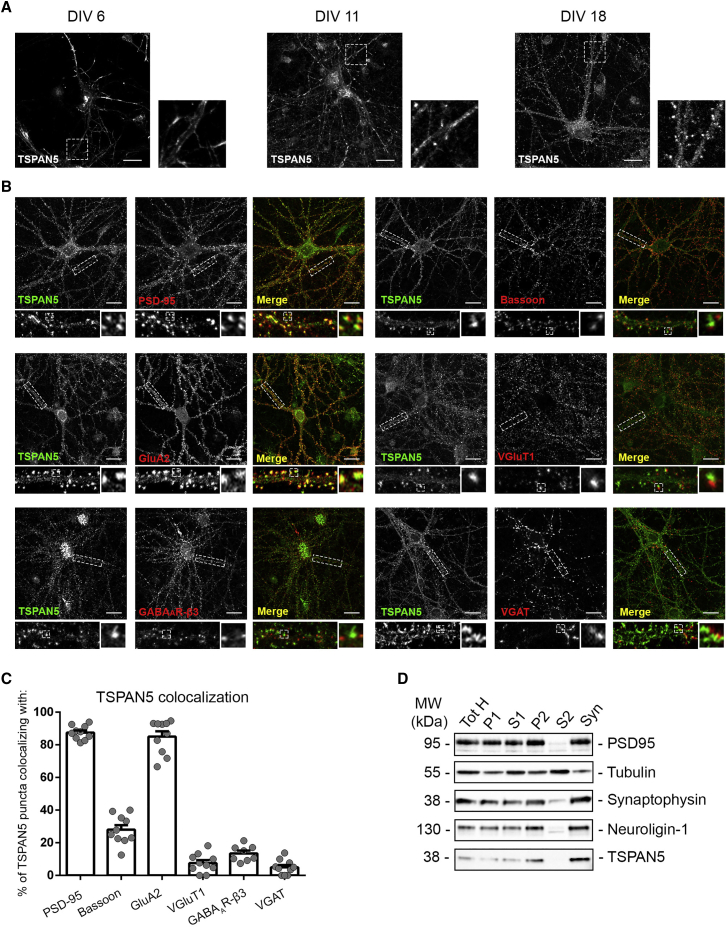
TSPAN5 Is Expressed in the Postsynaptic Compartment of Hippocampal Pyramidal Neurons (A) Confocal images of rat hippocampal cultured neurons immunolabeled for TSPAN5 at DIV 6, 11, and 18. Scale bar, 20 μm. Insets show higher magnification of regions highlighted in white. n = 3 independent cultures. (B) Confocal images of DIV 18 rat hippocampal cultured neurons immunolabeled for TSPAN5 (green) and the synaptic markers (red) PSD-95, GluA2, GABA_A_R-β3, Bassoon, VGluT1, and VGAT. Insets show higher magnification of regions highlighted in white. Scale bar, 20 μm. (C) Quantification of percentages of TSPAN5 puncta colocalizing with the different markers analyzed. n = 10 neurons. (D) Synaptosomes purification from adult rat hippocampi and cortices show TSPAN5 in the synaptosomal fraction. PSD-95, NLG1, and Synaptophysin are used as post- and presynaptic proteins, respectively. Tubulin is used as loading control. P, pellet; S, supernatant; Syn, synaptosomes; Tot H, total homogenate. Exact values are shown in Table S1. See also Figure S1. Graphs represent mean ± SEM.

To study TSPAN5 subcellular distribution, we immunostained cultured hippocampal pyramidal neurons at different maturation stages. In immature neurons, TSPAN5 distributed mostly on the dendrite edges (Figure 1A, day *in vitro* [DIV] 6 inset). At later stages, TSPAN5 localizes to filopodia and immature dendritic spines (Figure 1A, DIV 11 inset) as confirmed by the colocalization with N-cadherin, an early synaptic adhesion molecule (Figure S1E). At DIV 18, when neurons are generally considered mature (Barnes and Polleux, 2009, Li and Sheng, 2003), TSPAN5 is enriched in protrusions along dendrites, likely dendritic spines (Figure 1A, DIV 18 inset).

The postsynaptic localization of TSPAN5 in mature hippocampal neurons was confirmed by immunostaining (Figure 1B). We found a strong correlation between TSPAN5 distribution and excitatory postsynaptic markers (PSD-95, GLUA2/3), but poor overlap with presynaptic (Bassoon, excitatory: VGlut1; inhibitory: VGAT) and inhibitory postsynaptic (GABA_A_R-β3) markers (Figure 1C).

Accordingly, we also detected TSPAN5 in synaptosomes isolated from adult rat hippocampi and cortices homogenates (Figure 1D).

### TSPAN5 Regulates Dendritic Spine Formation

Postsynaptic localization of TSPAN5 at early stages points to a role in dendritic spine formation. To test this, we altered TSPAN5 levels by infecting cultured rat hippocampal neurons with lentiviral particles encoding scrambled shRNA (scrambled), shRNA targeting *Tspan5* (Sh-TSPAN5) or a bicistronic construct carrying Sh-TSPAN5 and human TSPAN5-GFP cDNA resistant to Sh-TSPAN5 (rescue). In Sh-TSPAN5-transduced neurons, a significant reduction of TSPAN5 expression was observed. The rescue significantly restored TSPAN5 levels, with partial cleavage splitting TSPAN5-GFP fusion protein (Figure S1F). We verified the knockdown of endogenous TSPAN5 both in the Sh-TSPAN5 and in the rescue conditions using real-time PCR with mouse-specific probes for *Tspan5* mRNA. No changes were found on other tetraspanin transcripts (TSPAN7 and CD81) (Figure S1G). TSPAN5 knockdown specificity was validated also by immunostaining on transfected rat hippocampal pyramidal neurons (Figure S1H).

**Figure 2:**
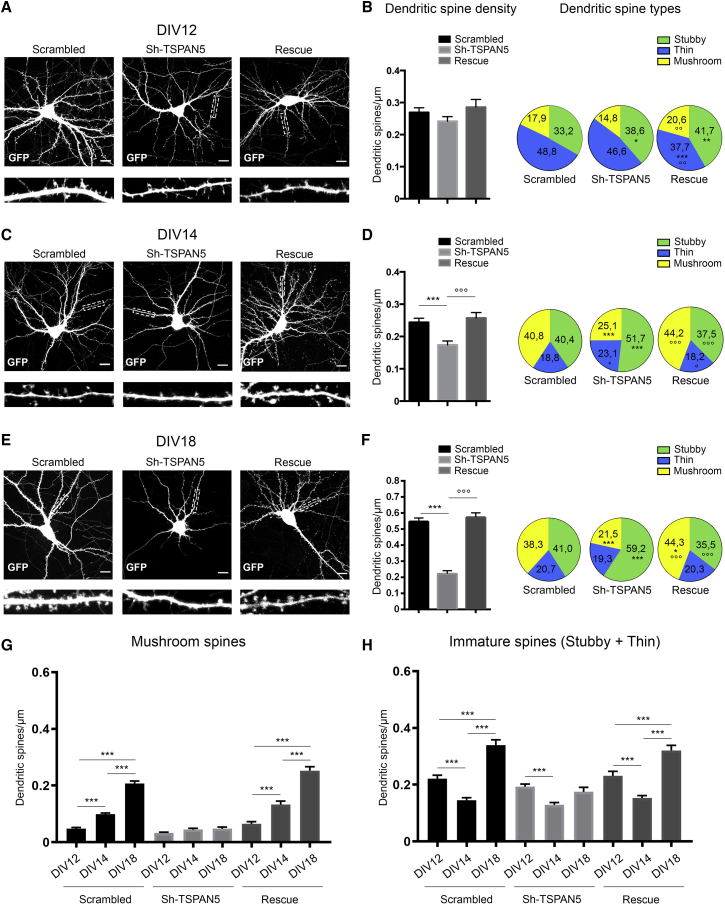
TSPAN5 Regulates Dendritic Spine Formation (A) Confocal images of DIV 12 rat hippocampal cultured neurons transfected at DIV 5 with scrambled, Sh-TSPAN5, or rescue constructs all co-expressing GFP. Insets show higher magnification of dendrites highlighted in white. Scale bar, 20 μm. (B) Left panels: quantification of dendritic spine density (dendritic spine/micrometer). Right panel: quantification of dendritic spine morphology presented as percentages of spines in three categories: stubby, thin, and mushroom (pie charts). Scrambled, n = 25 neurons; Sh-TSPAN5, n = 26 neurons; rescue, n = 22 neurons. (C) Confocal images of DIV 14 rat hippocampal cultured neurons transfected at DIV 5 with scrambled, Sh-TSPAN5, or rescue constructs all co-expressing GFP. Insets show higher magnifications of dendrites highlighted in white. Scale bar, 20 μm. (D) Left panels: quantification of dendritic spine density (dendritic spine/micrometer). Right panel: quantification of dendritic spine morphology shown as percentages of spines in three categories: stubby, thin, and mushroom (pie charts). Scrambled, n = 19 neurons; Sh-TSPAN5, n = 20 neurons; rescue, n = 20 neurons. (E) Confocal images of DIV 18 rat hippocampal cultured neurons transfected at DIV 5 with scrambled, Sh-TSPAN5, or rescue constructs all co-expressing GFP. Insets show higher magnification of dendrites highlighted in white. Scale bar, 20 μm. (F) Left panel: quantification of dendritic spine density (dendritic spine/micrometer). Right panel: quantification of dendritic spine morphology shown as percentages of spines in three categories: stubby, thin, and mushroom (pie charts). n = 16 neurons/condition. (G) Quantification of the density of mushroom spines from DIV 12, 14, and 18 neurons in (A), (C), and (E) transfected with scrambled, Sh-TSPAN5, or rescue constructs to highlight temporal changes (dendritic spine/micrometer). (H) Quantification of the density of immature dendritic spines (stubby and thin) from DIV 12, 14, and 18 neurons in (A), (C), and (E) transfected with scrambled, Sh-TSPAN5, or rescue constructs to highlight temporal changes (dendritic spine/micrometer). Exact values are shown in Table S1. See also Figure S1. Graphs represent mean ± SEM. ^∗^p < 0.05, ^∗∗^p < 0.01, and ^∗∗∗^p < 0.001 versus scrambled; °p < 0.05, °p < 0.01, and °p < 0.001 versus Sh-TSPAN5.

To evaluate the role of TSPAN5 in dendritic spine formation, we transfected cultured rat hippocampal neurons before synaptogenesis (DIV 5) for analysis at different maturation stages. At DIV 12 (Figure 2A), we detected no change in the density or morphology of dendritic spines (Figure 2B), except for an increase of the stubby spine count in the Sh-TSPAN5 condition (Figure 2B). In contrast, rescue-transfected neurons had significant changes across all dendritic spine types (Figure 2B). No defect was observed in dendritic branching (Figure S1I).

At DIV 14, synaptogenesis is prominent (Chanda et al., 2017); here we found a significant reduction in spine density upon TSPAN5 knockdown that was reversed in rescue-transfected neurons (Figures 2C and 2D). Notably, both scrambled- and rescue-transfected neurons at DIV 12 and 14 had a similar spine density, but the mushroom spine count doubled, confirming the physiological maturation of pre-existing immature spines in this time period (Figures 2D and 2G). This process was impaired in Sh-TSPAN5-transfected neurons, suggesting a role for TSPAN5 in spine maturation (Figures 2D, 2G, and 2H). In DIV 18 Sh-TSPAN5-transfected neurons, dendritic spine density was 60% reduced compared with the scrambled condition (Figures 2E and 2F). Most of the remaining spines were immature, thus the reduction in density is likely due to failure of maturation of spines that are then removed. These effects were reversed in the rescue-transfected neurons that also displayed a small increase in the percentage of mushroom spines, implying that increased TSPAN5 expression has a positive effect on spine maturation (Figure 2F).

We confirmed the excitatory nature of analyzed neurons by immunostaining for the excitatory marker CamKII-α (Benson et al., 1994) (Figure S1J).

### TSPAN5 Depletion Does Not Affect Excitatory Synapse Function

**Figure 3:**
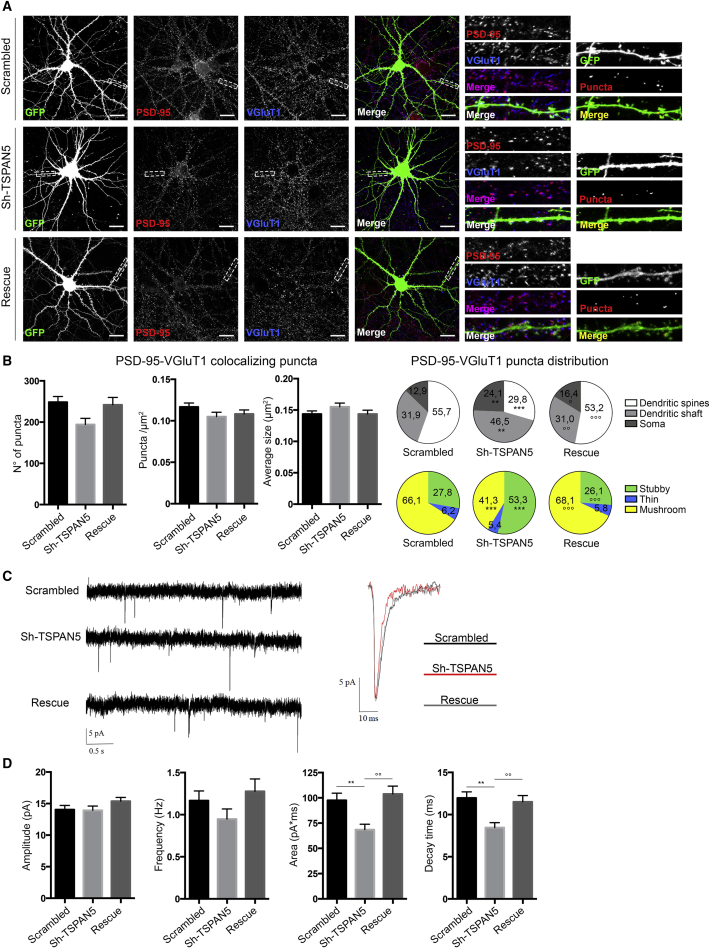
TSPAN5 Depletion Does Not Affect Excitatory Synapse Function (A) Confocal images of DIV 18 rat hippocampal cultured neurons transfected at DIV 5 with scrambled, Sh-TSPAN5, or rescue constructs all co-expressing GFP and immunolabeled for PSD-95 (red) and VGluT1 (blue). Insets show higher magnification of dendrites highlighted in white boxes and colocalizing puncta between PSD-95 and VGluT1 (cluster, red). (B) Quantification relative to (A) of PSD-95-VGluT1 colocalizing puncta in GFP positive areas (number of puncta), puncta density (puncta/square micrometer) and average size (square micrometers). Gray charts show percentage of puncta distributed among the dendritic spines (white), the dendritic shaft (light grey), and the soma (dark grey). Colored pie charts show the distribution of PSD-95-VGlut1 colocalizing puncta on different subtypes of dendritic spines (stubby, thin, and mushroom). n = 16 neurons/condition. (C) Representative traces of AMPAR-mediated mEPSCs recorded from DIV 18 rat hippocampal cultured neurons transfected at DIV 5 with scrambled, Sh-TSPAN5, or rescue constructs. Scrambled, n = 24; Sh-TSPAN5, n = 25; rescue, n = 19. (D) Quantification of amplitude, frequency, area, and decay time of AMPAR-mediated mEPSCs relative to (C). Exact values are shown in Table S1. See also Figure S2. Graphs represent mean ± SEM. ^∗^p < 0.05, ^∗∗^p < 0.01, and ^∗∗∗^p < 0.001 versus scrambled; °p < 0.05, °p < 0.01, and °p < 0.001 versus Sh-TSPAN5.

We further studied TSPAN5 and neuronal function by performing an immunostaining for the excitatory postsynaptic marker PSD-95 and the presynaptic marker VGluT1 on mature neurons (DIV 18) transfected before synaptogenesis with the scrambled, Sh-TSPAN5, or rescue construct (Figure 3A). Surprisingly, the number, density, and average size of the PSD-95-VGluT1 colocalizing puncta were unchanged in all three conditions, suggesting that excitatory synapses were unaffected by TSPAN5 modulation (Figure 3B).

In pyramidal neurons most excitatory synapses form on dendritic spines, but previous work has shown excitatory synapses on dendritic shafts in physiological conditions both *in vitro* and *in vivo* (Harris et al., 1992, Trachtenberg et al., 2002). The proportion of shaft synapses varies during neuronal maturation (Harris et al., 1992) and can be affected by genetic manipulation of proteins involved in dendritic spine formation (Aoto et al., 2007, Lee et al., 2016, Niesmann et al., 2011).

Thus, we analyzed the distribution of excitatory synapses formed on dendritic spines, dendritic shafts, and the soma. As expected, the fraction of PSD-95-VGluT1 colocalizing puncta on dendritic spines decreased in Sh-TSPAN5 condition compared with scrambled controls, with a parallel increase of puncta number on both the dendritic shaft and soma. This was reversed in the rescue condition (Figure 3B, gray pie charts).

When analyzing the morphology of dendritic spines positive for PSD-95-VGluT1 colocalizing puncta, TSPAN5-knockdown neurons exhibited a greater proportion of puncta on stubby spines and smaller on mushroom spines compared with both scrambled and rescue conditions (Figure 3B, colored pie charts), reinforcing the idea that TSPAN5 promotes morphological maturation to mushroom-shaped dendritic spines, regardless of synapses.

Next, we verified synapse functionality by electrophysiological recordings of AMPAR-mediated miniature excitatory postsynaptic currents (mEPSCs). Upon TSPAN5 modulation, the frequency and amplitude of mEPSCs were not significantly changed (Figures 3C and 3D), similar to other studies in which the number of shaft synapses was increased (Aoto et al., 2007, Lee et al., 2016, Niesmann et al., 2011). However, the area and decay time of currents were decreased in Sh-TSPAN5-transfected neurons (Figures 3C and 3D), suggesting a change in the composition of AMPAR subunits (Henley and Wilkinson, 2016) or auxiliary subunits (Greger et al., 2017). The ratio between amplitudes of AMPAR- and N-methyl-D-aspartate receptor (NMDAR)-mediated currents (Figure S2A), as well as miniature inhibitory postsynaptic currents (mIPSCs) (Figure S2B), were unchanged by TSPAN5 modulation.

We also tested presynaptic function by exposing transfected neurons to high sucrose concentration (1 M) to trigger presynaptic vesicle release (Antonucci et al., 2013). No change in excitatory currents were detected, implying that presynaptic function is unaffected by TSPAN5 knockdown (Figure S2C).

Furthermore, upon TSPAN5 knockdown, the density of the VGlut1 puncta was reduced, while their average size increased (Figure S2D), suggesting functional compensation of these two effects.

### TSPAN5 Interacts with NLG1

Because tetraspanins are known to form TEMs, we wondered if TSPAN5 can organize similar structures in neurons and whether TEMs are involved in dendritic spine maturation.

TEMs present a characteristic lipidic composition (Yáñez-Mó et al., 2009) that determines their solubilization properties: TEMs are solubilized by strong detergents (e.g., Triton X-100), but they precipitate with digitonin treatment because of its binding to cholesterol (Charrin et al., 2003a, Yáñez-Mó et al., 2009). Lysis of cultured hippocampal neurons during synaptogenesis (DIV 12) in a buffer containing Triton X-100 and NP40 (RIPA buffer) revealed an enrichment of TSPAN5 in the supernatant, whereas digitonin led to its concentration in the pellet (Figure S3A). This result supports the presence of TSPAN5 TEMs in immature neurons.

To investigate if these domains are key for dendritic spine formation, we examined the presence in TEMs of NLG1 and the AMPAR subunit GluA2, two crucial players in dendritic spine formation and excitatory synapses function, respectively (Bassani et al., 2009, Bassani et al., 2013, Chih et al., 2005, Hall and Ghosh, 2008). Both proteins were detected in the same fractions as TSPAN5, indicating their possible presence in TEMs (Figure S3A).

However, this method is not specific for TEMs, as digitonin precipitates all cholesterol-enriched domains (e.g., lipid rafts), which can be found in dendritic spines (Hering et al., 2003).

**Figure 4:**
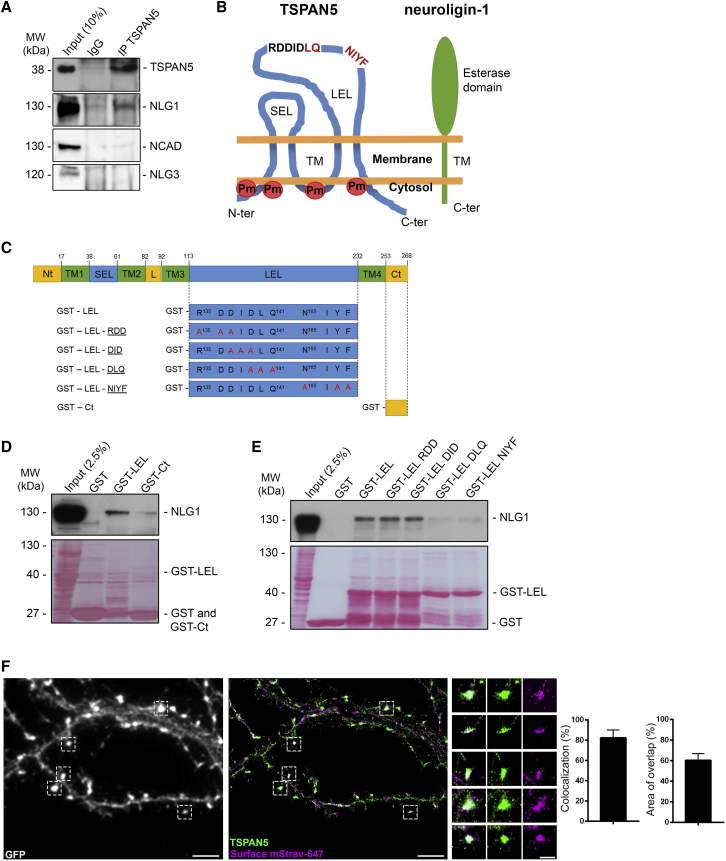
TSPAN5 Interacts with NLG1 through the LEL Domain (A) Co-immunoprecipitation experiments of adult rat hippocampus and cortex lysates in RIPA buffer. Input: 10% of immunoprecipitated volume. Immunoprecipitation: α-rabbit IgG or α-TSPAN5 antibody. Blots probed for TSPAN5, NLG1, N-cadherin (NCAD), and neuroligin-3 (NLG3). (B) Schematic of TSPAN5 (blue) and NLG1 (green) structure. TSPAN5 structure inferred by homology with other tetraspanins. C-ter, C terminus; LEL, large extracellular loop; N-ter, N terminus; SEL, small extracellular loop; TM, transmembrane regions. The R^135^DDIDLQ^141^ and N^165^IYF^168^ sequences of LEL are shown; residues involved in NLG1 interaction are indicated in red. Palmitoylation (Pm) sites mutated in the PLM (Figure 6D) are highlighted in red. Plasma membrane shown in orange. (C) Schematic of GST constructs used in pull down experiments. TSPAN5 domains are highlighted: in yellow, intracellular domains (Ct, C-terminal; L, loop; Nt, N-terminal); in green, transmembrane domains (TM1–TM4); and in blue, extracellular domains (LEL, large extracellular loop; SEL, small extracellular loop). Constructs with mutated residues highlighted in red. (D) GST pull down experiments in adult rat hippocampus and cortex lysates using empty GST, GST fused to LEL (GST-LEL), or GST fused to the C terminus (GST-Ct). The input was 2.5% of the pull down volume. Blots probed for NLG1. Red Ponceau shows GST-bound fragments. (E) GST pull down experiment in adult rat hippocampus and cortex lysates using empty GST, GST-LEL, or GST fused to LEL with different mutations: RDD, DID, DLQ, and NIYF. The input was 2.5% of the pull down volume. Blots probed for NLG1. Red Ponceau shows the GST-bound fragments. (F) dSTORM images of DIV 12 hippocampal neurons transfected with sh-Nlg1 (expressing GFP), AP-NLG1, and BirA-ER surface labeled with mStrav-647 (purple) and labeled after fixation and permeabilization with α-TSPAN5 antibody (green). Scale bar, 4 μm. Right images show higher magnification of regions highlighted in white; scale bar, 1 μm. Histograms show percentage of TSPAN5 puncta colocalizing with AP-NLG1 and percentage of area overlap. n = 4 neurons. Exact values are shown in Table S1. See also Figure S3. Graphs represent mean ± SEM.

Thus, we performed co-immunoprecipitation experiments in RIPA lysates from adult rat hippocampi and cortices, confirming the association of TSPAN5 with NLG1 (Figure 4A). We did not detect other postsynaptic adhesion molecules, such as N-cadherin and NLG3 in TSPAN5 immunoprecipitates (Figure 4A).

Importantly, this interaction is not mediated by indirect association between different tetraspanins in TEMs, because the experiments were performed in the presence of Triton X-100 and NP40 in the lysis buffer (Hemler, 2001).

### The TSPAN5-NLG1 Interaction Is Mediated by the LEL Domain

To map the TSPAN5-NLG1 interaction, we analyzed the main functional domains of tetraspanins, the surface exposed LEL and the C terminus (Ct), exposed to the cytosol (Figure 4B).

We produced GST-LEL or GST-Ct fusion proteins (Figure 4C) for pull down experiments using lysates from adult rat hippocampi and cortices. We clearly detected NLG1 with GST-LEL; conversely, there was a weak signal with GST-Ct (Figure 4D). We failed to detect GluA2/3 in GST-LEL pull down (Figure S3B).

Consequently, we focused on the interaction between the LEL domain of TSPAN5 and NLG1. On the basis of previous work on the TSPAN5 LEL domain (Saint-Pol et al., 2017), we hypothesized that two motifs specific to the C8 tetraspanin subgroup could mediate this interaction (Figures 4B and 4C). We used LEL domains with these motifs mutated to alanines (Figure 4C) in GST pull down experiments and found that the NLG1 interaction was dependent on the D^139^LQ^141^ and N^165^IYF^168^ regions (Figure 4E).

We confirmed this association in HEK293 cells co-transfected with full-length TSPAN5-GFP (either wild-type or DLQ or NIYF mutants) together with AP-NLG1 and BirA-ER. The AP tag, on the extracellular side of NLG1, is biotinylated by the enzyme BirA-ER in the ER (Chamma et al., 2016a, Howarth et al., 2006). We used streptavidin coupled to agarose beads to pull down biotinylated AP-NLG1 and found that it coprecipitated wild-type TSPAN5-GFP, while the DLQ and NIYF mutants were barely detectable (Figure S3C).

We also verified the involvement of the TSPAN5 Ct in NLG1 binding. When the Ct was truncated (ΔC), there was little precipitation of TSPAN5 compared with wild-type TSPAN5 (Figure S3D), and no co-precipitation was detected with the double mutants DLQ-ΔC and NIYF-ΔC.

We also analyzed the region of NLG1 interacting with TSPAN5. We transfected HEK293 cells with TSPAN5-GFP and with HA-tagged wild-type NLG1, or NLG1 mutants with either the extracellular cholinesterase-homology region replaced with that of human acetylcholinesterase (SWAP mutant; Scheiffele et al., 2000) or where the final 72 aa of the Ct were truncated (ΔC) (Figures 4B and S3E). We detected a robust interaction with wild-type or the ΔC mutant but a weak association with the SWAP mutant, suggesting that the extracellular region of NLG1 is responsible for TSPAN5 interaction.

Finally, we determined the localization of this interaction by dSTORM super-resolution imaging using DIV 12 rat hippocampal neurons transfected with shNLG1 and expressing AP-NLG1 and BirA-ER (Figure 4F). We labeled surface AP-NLG1 in live neurons and immunostained endogenous TSPAN5 after fixation and permeabilization. We found high colocalization of the two signals mainly in regions protruding from dendrites (Figure 4F), suggesting that the association occurs on the surface membrane of maturing dendritic spines.

### TSPAN5-NLG1 Interaction Is Vital for Dendritic Spine Maturation

NLG1 is known to play many roles in synapse differentiation and maturation of dendritic spines, but its precise contribution is still being investigated (Chanda et al., 2017, Chih et al., 2005, Kwon et al., 2012, Letellier et al., 2018, Wu et al., 2019). Thus, we explored if the TSPAN5-NLG1 interaction is involved in these processes.

**Figure 5:**
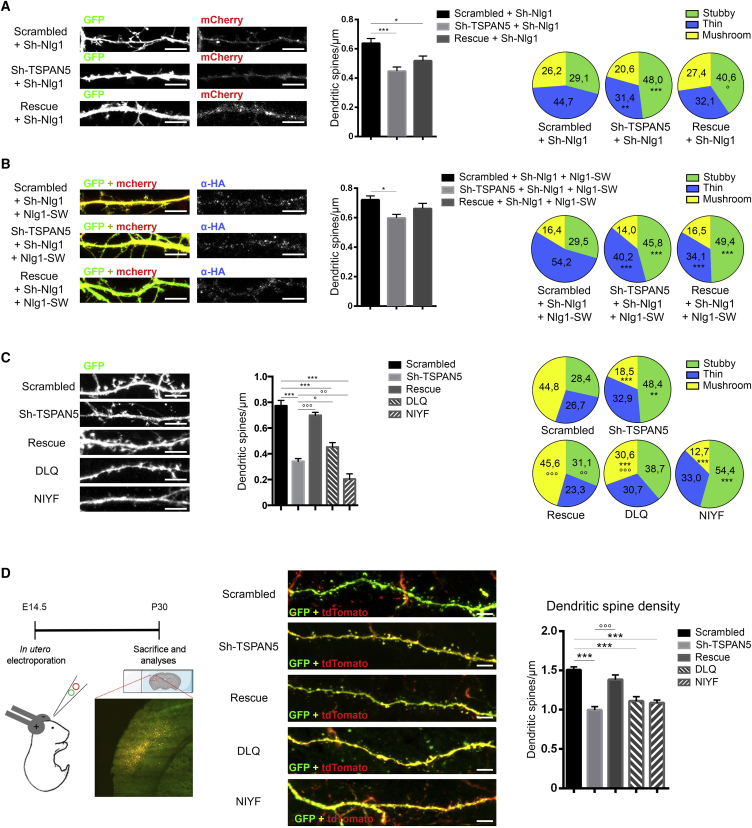
TSPAN5-NLG1 Interaction Is Crucial for Dendritic Spine Maturation (A) Confocal images of DIV 18 rat hippocampal cultured neurons transfected at DIV 5 with a GFP co-expressing Sh-Nlg1 and either scrambled, Sh-TSPAN5, or rescue constructs all co-expressing mCherry. Scale bar, 5 μm. Quantification of dendritic spine density (dendritic spine/micrometer). Pie charts show dendritic spine morphology as percentages of spines in three categories: stubby, thin, and mushroom. Scrambled, n = 17 neurons; Sh-TSPAN5, n = 19 neurons; rescue, n = 15 neurons. (B) Confocal images of DIV 18 rat hippocampal cultured neurons transfected at DIV 5 with a GFP co-expressing Sh-Nlg1, HA-tagged NLG1 SWAP mutant (Nlg1-SW), and scrambled, Sh-TSPAN5, or rescue constructs all co-expressing mCherry and immunolabeled for HA (blue). Scale bar, 5 μm. Quantification of dendritic spine density (dendritic spine/micrometer). Pie charts display dendritic spine morphology as percentages of spines in three categories: stubby, thin, and mushroom. Scrambled, n = 19 neurons; Sh-TSPAN5, n = 17 neurons; rescue, n = 17 neurons. (C) Confocal images of DIV 18 rat hippocampal cultured neurons transfected at DIV 5 with scrambled, Sh-TSPAN5, rescue, DLQ, or NIYF constructs all co-expressing GFP. Scale bar, 5 μm. Quantification of dendritic spine density (dendritic spine/micrometer). Pie charts show dendritic spine morphology as percentages of spines in three categories: stubby, thin, and mushroom. Scrambled, n = 16 neurons; Sh-TSPAN5, n = 14 neurons; rescue, n = 16 neurons; DLQ, n = 16 neurons; NIYF, n = 16 neurons. (D) In utero electroporation experiment. Left panel: experiment timeline schematic. E13.5 mice embryos were electroporated with pCAG-tdTomato and scrambled, Sh-TSPAN5, rescue, DLQ, or NIYF constructs all co-expressing GFP and perfused at P30 for imaging. Central panel: confocal images of secondary dendrites of cortical pyramidal neurons. Scale bar, 5 μm. Right panel: quantification of the density of dendritic spines (dendritic spines/micrometer). Scrambled, n = 44 dendrites; Sh-TSPAN5, n = 45 dendrites; rescue, n = 37 dendrites; DLQ, n = 45 dendrites; NIYF, n = 40 dendrites from three animals per condition. Exact values are shown in Table S1. Graphs represent mean ± SEM. ^∗^p < 0.05, ^∗∗^p < 0.01, and ^∗∗∗^p < 0.001 versus Scrambled; °p < 0.05, °p < 0.01, and °p < 0.001 versus Sh-TSPAN5.

First, we altered TSPAN5 levels in neurons co-transfected with a shRNA for NLG1 (Sh-Nlg1) (Chih et al., 2005) (Figure 5A). As expected, in neurons transfected with Sh-Nlg1 and scrambled for TSPAN5, the proportion of mushroom spines was reduced in favor of thin spines compared with neurons with normal NLG1 expression (compare Figure 5A with Figure 2F or 5C).

The proportion of mushroom spines did not change between scrambled- and Sh-TSPAN5-transfected neurons (Figure 5A), supporting the idea that both TSPAN5 and NLG1 act on the same maturation pathway. However, also in the absence of NLG1, the spine count reduced upon TSPAN5 knockdown (Figure 5A). In addition, TSPAN5 knockdown promoted a higher proportion of stubby spines and a smaller number of thin spines; this may be due to other NLG1-independent function(s) of TSPAN5 or incomplete NLG1 knockdown.

We validated this with a HA-tagged NLG1 SWAP mutant (Figure 5B). As expected by the reduced interaction with TSPAN5 (Figure S3C), this mutant did not rescue the effects of NLG1 knockdown, and the concomitant TSPAN5 knockdown had no additive effects on the proportion of mushroom spines (Figure 5B). As in the previous experiment, TSPAN5 knockdown induced a small reduction in dendritic spine density and an increase in stubby spines at the cost of a reduction in thin spines.

To verify the importance of TSPAN5-NLG1 interaction, we compared dendritic spines of neurons transfected with scrambled, Sh-TSPAN5 or rescue constructs, expressing either wild-type TSPAN5 (rescue) or the DLQ or NIYF mutants (Figure 5C). First, we confirmed that TSPAN5 encoded by the rescue constructs (rescue, DLQ or NIYF) was able to reach the neuronal surface by BS3 crosslinking experiments in neurons (Figure S3F).

The TSPAN5 mutants, unable to interact with NLG1, did not rescue TSPAN5 knockdown-mediated loss of dendritic spines (Figure 5B). Furthermore, the DLQ mutant caused an intermediate spine density between scrambled and Sh-TSPAN5-transfected neurons (Figure 4E). The NIYF mutant evoked stronger effects than the TSPAN5 knockdown alone (Figure 5C), which may be due to its partial ER retention that could induce ER stress and/or toxicity, as previously suggested (Saint-Pol et al., 2017).

Similar results were obtained when we examined the morphology of the remaining spines. Neurons transfected with the DLQ mutant had fewer mushroom spines compared with scrambled-transfected neurons but higher than the Sh-TSPAN5-transfected neurons with a co-occurring increase in stubby spines. The NIYF-transfected neurons instead exhibited spine morphology similar to the Sh-TSPAN5 condition (Figure 5C).

To confirm our results *in vivo*, we analyzed dendritic spines in brain slices from adult mice (postnatal day [P] 30) that had undergone in utero electroporation of these constructs at embryonic day (E) 13.5. We first validated the shRNA for TSPAN5 in mouse neuronal cultures (Figure S3G).

We analyzed secondary branches of the apical dendrite of transfected pyramidal cortical neurons from layer III in the somatosensory cortex and found a significant decrease in dendritic spine density upon transfection with Sh-TSPAN5 or DLQ and NIYF mutants compared with scrambled and rescue constructs. However, the magnitude of these effects was smaller than that observed in cultured neurons, possibly because of compensatory mechanisms occurring *in vivo* (Figure 5D).

### TSPAN5 Promotes NLG1 Clustering

Next, we studied how TSPAN5-NLG1 interaction regulates NLG1 function.

Previous work has shown that TSPAN5 interaction with ADAM-10 regulates its intracellular trafficking and ER exit (Saint-Pol et al., 2017). However, as shown by BS3 crosslinking experiments on neurons at DIV 12, TSPAN5 silencing has no effect on NLG1 trafficking (Figure S4A).

Because NLG1 clustering at sites of maturing dendritic spines is key for spine development (Dean et al., 2003), we investigated if TSPAN5 was part of this process.

**Figure 6:**
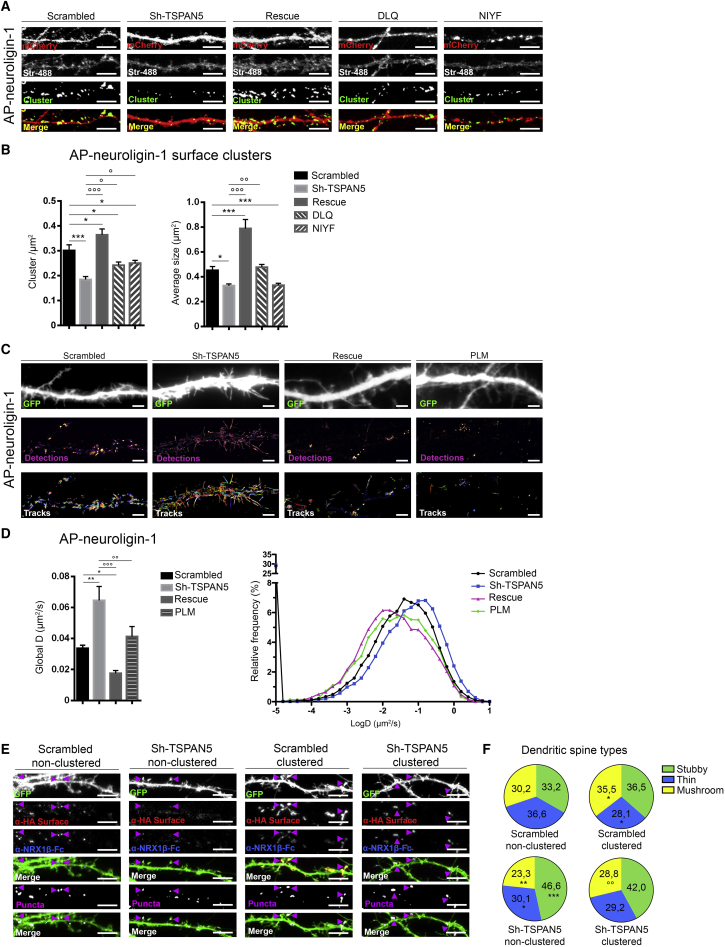
TSPAN5 Promotes NLG1 Clustering (A) Confocal images of dendrites from DIV 12 rat hippocampal cultured neurons transfected at DIV 5 with AP-NLG1, BirA-ER, and scrambled, Sh-TSPAN5, rescue, DLQ, or NIYF all co-expressing mCherry. Surface-applied Streptavidin-488 clusters shown in green. Scale bar, 5 μm. (B) Quantification of the cluster density (clusters/square micrometer) and average size (square micrometers) of Streptavidin-488 clusters. Scrambled, n = 19 neurons; Sh-TSPAN5, n = 20 neurons; rescue, n = 18 neurons; DLQ, n = 27 neurons; NIYF, n = 27 neurons. (C) Single-molecule tracking experiment with mStrav-647. Images of DIV 12 rat hippocampal cultured neurons transfected at DIV 5 with AP-NLG1, BirA-ER, and scrambled, Sh-TSPAN5, rescue, or PLM all co-expressing GFP. Top panels: widefield GFP signal of dendrites. Middle panels (intensity): super-resolved mStrav-647 detection maps as an intensity scale. Bottom panels (tracks): mStrav-647 trajectories shown in pseudocolors. Scale bar, 2 μm. (D) Quantification of single-molecule tracking. Left panel: quantification of global diffusion coefficient (square micrometers per second). Right panel: logarithmic distribution plot of diffusion coefficients of scrambled- (black), Sh-TSPAN5- (blue), rescue- (purple), or PLM-transfected neurons (green). Scrambled, n = 16 neurons; Sh-TSPAN5, n = 17 neurons; rescue, n = 16 neurons; PLM, n = 12 neurons. (E) Confocal images of DIV 14 cultured rat hippocampal neurons transfected at DIV 5 with HA-tagged NLG1 and either GFP-expressing scrambled or Sh-TSPAN5 constructs. Neurons treated from DIV 12 to 14 with either non-clustered neurexin1β-Fc (left panels) or neurexin1β-Fc pre-clustered with an unlabeled α-Fc antibody (right panels). Surface-applied α-HA antibody shown in red; surface-applied Alexa 647-labeled α-Fc shown in blue. Purple arrowheads show mushroom spines positive for clusters. (F) Pie charts of the proportion of stubby, thin, or mushroom spines relative to (E). Exact values are shown in Table S1. See also Figures S4–S7. Graphs represent mean ± SEM. In (F), ^∗^p < 0.05, ^∗∗^p < 0.01, ^∗∗∗^p < 0.001 versus scrambled non-clustered; °p < 0.05, °p < 0.01, °p < 0.001 versus Sh-TSPAN5 non-clustered.

Despite the global increase in spine density due to NLG1 overexpression (Letellier et al., 2018, Levinsoni et al., 2005), AP-tagged NLG1 overexpression did not modify the proportion of mushroom spines at DIV 12 or DIV 18 (Figures S4B and S4C compared with Figure 2). Thus, we transfected neurons before synaptogenesis with AP-NLG1, BirA-ER, and either mCherry-tagged scrambled, Sh-TSPAN5, rescue, DLQ, or NIYF constructs for analysis at DIV 12 of surface-expressed AP-NLG1 by using streptavidin-Alexa Fluor 488 (Figures 6A and 6B).

Analysis of clusters revealed a significant decrease in puncta density and their average size in the Sh-TSPAN5-transfected neurons compared with scrambled controls. This effect was reversed in rescue condition. Similarly to what happened to dendritic spines, expression of the DLQ mutant partially rescued this effect, whereas NIYF failed to do so (Figures 6A and 6B). These defects were detected at DIV 12, when dendritic spine density and morphology appear unaffected; this suggests this molecular defect to be responsible for the inhibition of dendritic spine maturation.

As a control, we analyzed the surface clustering of overexpressed SEP-N-cadherin (Bian et al., 2015) and HA-NLG3 (Figures S5A and S5B) given their lack of interaction with TSPAN5 (Figure 4A). As expected, TSPAN5 knockdown did not affect N-cadherin cluster density or size. On the other hand, a small but significant increase in NLG3 cluster size was detected (Figure S5B), possibly due to a partial compensatory mechanism, as NLG1 and NLG3 are known to share some functions (Chanda et al., 2017) or because of the heterodimerization of NLG1 and NLG3 (Poulopoulos et al., 2012).

Because NLG1 membrane clustering is crucial for neurexin clustering at presynaptic sites (Dean et al., 2003), we analyzed neurexin distribution upon TSPAN5 knockdown with a pan-neurexin-1 antibody (Figure S6). As expected by the low number of synapses at DIV 12, clustering of neurexin-1 was not affected by altering TSPAN5 (Figure S6A). A small reduction in cluster density was detected, resembling in magnitude that seen for dendritic spine density and the proportion of mushroom spines (Figures 2A and 2B).

In DIV 18 neurons, TSPAN5 knockdown affected both neurexin-1 cluster density and the staining intensity (Figure S6B). All effects were reversed by expression of rescue construct (Figure S6B).

Recently it was shown that upon neuronal maturation, NLG1 surface mobility is reduced, and it tends to aggregate in confined areas (Chamma et al., 2016a, Chamma et al., 2016b, Chamma et al., 2017).

We hypothesized that TSPAN5 could be involved in this process by slowing NLG1 mobility inside TEMs, promoting its accumulation in clusters and binding to neurexin.

We performed single-molecule tracking experiments on overexpressed AP-NLG1 using monomeric streptavidin coupled to Atto-647 (Chamma et al., 2016a, Chamma et al., 2016b, Chamma et al., 2017) in neurons at DIV 12 transfected with scrambled, Sh-TSPAN5, or rescue construct (Figure 6C).

Sh-TSPAN5-transfected neurons showed an increased NLG1 diffusion compared with scrambled (Figure 6D), indicating that TSPAN5 slows down NLG1 mobility. Conversely, rescue-transfected neurons showed a decrease in these parameters at levels even lower than the scrambled condition (Figures 6C and 6D).

We tested a TSPAN5 palmitoylation-defective mutant (PLM) in which the four cysteines (Cys 13, 14, 80, and 252) in the juxta-membrane region were mutated to alanines (palmitoylation [Pm] sites in Figure 4B) (Dornier et al., 2012). We verified that the PLM interacts with NLG1 (Figure S7A) and reaches the plasma membrane (Figure S7B).

The PLM is predicted to retain the ability to organize TSPAN5 TEMs but would be unable to interact with other tetraspanins preventing higher order association (Charrin et al., 2002, Dornier et al., 2012, Espenel et al., 2008, Hemler, 2001, Yang et al., 2002, Zuidscherwoude et al., 2015). Co-expression of the Sh-TSPAN5 and PLM-TSPAN5-GFP mutant restored the global AP-NLG1 diffusion coefficient to levels similar to those of scrambled condition but lower than rescue-transfected neurons (Figure 6D). This suggests that homotypic TSPAN5 domains are responsible for building a membrane scaffold for dendritic spine formation and that higher level tetraspanin interactions further enhance this function (Figures 6C and 6D).

As a control, we measured the surface mobility of N-cadherin tagged with the photoconvertible protein mEOS2 (Garcia et al., 2015) and found no change of diffusion coefficients (Figure S7C).

We also evaluated the mobility of the AMPAR subunit GluA2 to study if impaired NLG1 clustering upon TSPAN5 disruption could affect accumulation of neurotransmitter receptors (Figure S7D). Single-molecule tracking performed using uPAINT with α-GluA2 antibodies coupled to Atto-647 (Nair et al., 2013) on DIV 12 neurons showed that GluA2 had significantly higher diffusion coefficients upon TSPAN5 knockdown. This effect was abolished in the rescue condition (Figure S7D). We hypothesize that this is due to reduced NLG1 cluster density and size (Figures 6A and 6B), supporting previous work describing the role of NLG1 mobility in AMPAR clustering (Letellier et al., 2018, Mondin et al., 2011).

### NLG1 Artificial Clustering Blocks the Effects of TSPAN5 Knockdown

Finally, we attempted to revert changes caused by TSPAN5 knockdown by driving NLG1 clustering. Thus, we incubated DIV 12 neurons for 48 h with exogenous neurexin1β-Fc, either pre-clustered by an α-Fc antibody (clustered condition) or not (non-clustered condition) (Mondin et al., 2013) (Figures 6E, 6F, and S7E).

We verified that adding clustered neurexin1β-Fc increased the average size of surface HA-NLG1/neurexin1β-Fc puncta without affecting dendritic spine density (Figure S7E).

We found that non-clustered Sh-TSPAN5-transfected neurons still had a reduced proportion of mushroom spines compared with non-clustered scrambled-transfected neurons (Figures 6E and 6F). Treatment with clustered neurexin1β-Fc increased the proportion of mushroom spines both in scrambled and Sh-TSPAN5-transfected neurons indicating a partial rescue in dendritic spine maturation (Figure 6F). Previous work has shown that inducing NLG1 clustering is sufficient to promote the formation of mushroom-like protrusions (Mondin et al., 2011, Mondin et al., 2013). Because the same effect was seen in TSPAN5 knockdown, we conclude that the role of TSPAN5 in dendritic spine maturation occurs by a direct regulation of NLG1 clustering.

## Discussion

Overall, our data indicate that TSPAN5 is a regulator of NLG1 clustering. We found this process to control dendritic spine maturation without affecting the general assembly of postsynaptic compartments. Our findings are a proof of principle that compartmentalization of transmembrane proteins through tetraspanins is an extra level of regulation of dendritic spine formation.

### TSPAN5 TEMs Regulates NLG1 Clustering

We characterized TSPAN5 localization and function, finding that it binds NLG1 through the LEL domain. This domain is key for many tetraspanin interactions (Hemler, 2005), and it was previously found to bind ADAM-10 via the residues R^135^DD^137^ and N^165^IYF^168^. Together with our data, this shows that the R^135^DD^137^ and D^139^LQ^141^ sequences specifically regulate the association of TSPAN5 LEL with ADAM-10 and NLG1, respectively. In contrast, the N^165^IYF^168^ motif is involved in multiple TSPAN5 interactions. TSPAN5-ADAM-10 interaction regulates the intracellular trafficking of the protease and the cleavage of specific targets (Dornier et al., 2012, Haining et al., 2012, Noy et al., 2016, Saint-Pol et al., 2017), such as Notch and CD44 (Jouannet et al., 2016), transmembrane proteins implicated in dendritic spine plasticity (Bijata et al., 2017, Roszkowska et al., 2016). NLG1 also undergoes activity-dependent proteolytic cleavage by ADAM-10 in neurons (Suzuki et al., 2012). However, we did not detect a change in the amount of full-length NLG1 upon TSPAN5 modulation.

The TSPAN5-NLG1 interaction appears to be tripartite: two sites are specific for NLG1 (DLQ and C-terminal tail), and another is shared with ADAM-10 (NIYF) (Saint-Pol et al., 2017). The model based on our results predicts that the DLQ sequence is necessary for TSPAN5-NLG1 binding and the presence of either the NIYF site or the C-terminal tail is also needed. DLQ and NIYF sequences are shared among the C8 subgroup of tetraspanins (Saint-Pol et al., 2017). Thus, other members of this subgroup could interact with NLG1, although the TSPAN5 Ct has sequence homology only with TSPAN14 (60% identity) and TSPAN17 (86.6% identity).

TSPAN5 has a vital role in NLG1 clustering, trapping it in TEMs and regulating its surface mobility. This phenomenon is NLG1 specific, as there was no change in N-cadherin mobility upon TSPAN5 modulation. Interestingly, super-resolution analyses have shown that the average dimension of TEMs (between 90 and 190 nm) (Nydegger et al., 2006, Termini et al., 2014, Zuidscherwoude et al., 2015) and NLG1 clusters (98 nm) (Chamma et al., 2016a) are similar. A related trapping mechanism was observed for CD81, shown to reduce CD19 mobility in B cells (Mattila et al., 2013).

Moreover, the defects in surface mobility of the GluA2 subunit of AMPARs upon TSPAN5 knockdown suggest that reducing NLG1 clustering impairs recruitment of other postsynaptic components (Chanda et al., 2017, Letellier et al., 2018, Mondin et al., 2011). Alternatively, AMPARs may be directly trapped in TSPAN5 TEMs; however, this is unlikely, as we have shown that TSPAN5-GluA2 association was not dependent on the LEL.

### TSPAN5 Regulates Dendritic Spine Maturation

Our data found that TSPAN5 regulates dendritic spine maturation more than formation. Moreover, TSPAN5 downregulation did not affect the total number of excitatory synapses, because of an increase in shaft synapses.

It has been observed both *in vitro* and *in vivo* that modulation of proteins involved in dendritic spine morphology, such as EphrinB3, nArgBP2 and Neurobeachin can lead to changes in the number of shaft excitatory synapses with no dramatic effect on mEPSCs (Aoto et al., 2007, Lee et al., 2016, Niesmann et al., 2011).

We hypothesize that this is a compensatory effect to maintain normal excitatory currents. An alternative proposed (Harris et al., 1992) is that under physiological conditions, shaft excitatory synapses can form at early stages of neuronal maturation, and dendritic spines are built at the same site following the Miller/Peters model (Yuste and Bonhoeffer, 2004). The observed failure in dendritic spine maturation in the absence of TSPAN5 may lead to the maintenance of shaft synapses.

The role of NLG1 in dendritic spine and synapse formation remains controversial. Earlier reports showed its direct role in synapse formation, yet later work argued that NLG1 participates only in the maturation of synapses (Chih et al., 2005, Chubykin et al., 2005, Chubykin et al., 2007, Kwon et al., 2012, Prange et al., 2004, Scheiffele et al., 2000, Wu et al., 2019). The normal number of excitatory synapses and the absence of overt defects in PSD-95-VGluT1 cluster number and size upon TSPAN5 knockdown suggest that NLG1 surface mobility and clustering have specific functions in the maturation of dendritic spines without impairing excitatory synapses. This is surprising considering that the PDZ domain of NLG1 binds PSD-95, the main postsynaptic density scaffold protein (Mondin et al., 2011, Nam and Chen, 2005).

The lack of effect on excitatory synapse number may be due to compensation of other adhesion molecules that bind PSD-95 (Han and Kim, 2008), or by the ability of PSD-95 to bind membranes (Topinka and Bredt, 1998). Recently, a role for NLG1 in inducing dendritic spine formation by SPAR binding and activation of LIMK1/cofilin-dependent actin remodeling was found (Liu et al., 2016). Perhaps the disruption of NLG1 clustering upon TSPAN5 knockdown impairs this function, thus affecting actin remodeling which is crucial for dendritic spines.

Notably, the knockdown of NLG1 alone was found to reduce the number of dendritic spines and only slightly affect AMPAR-mediated mEPSCs or evoked excitatory postsynaptic currents (eEPSCs) (Chanda et al., 2017, Chih et al., 2005) similar to our results upon TSPAN5 knockdown. This may be due to partial compensation by NLG3 that is unaffected by TSPAN5 modulation and can localize to excitatory synapses (Budreck and Scheiffele, 2007). Indeed, the knockdown of NLG1 and NLG3 was reported to cause strong defects in AMPAR-mediated currents (Chanda et al., 2017).

NLG1 has been shown to have a specific regulatory role on NMDAR-mediated currents (Kwon et al., 2012, Wu et al., 2019), but these are not impaired by TSPAN5 knockdown. Our results suggest that this function is not dependent on NLG1 clustering or that the remaining clusters are still able to promote physiological NMDAR recruitment. Alternatively, compensatory mechanisms, possibly mediated by NLG3 or other NMDAR interactors, may occur.

Our work does not exclude other processes underlying dendritic spine defects upon TSPAN5 knockdown; however, TSPAN5 silencing in neurons in which NLG1 was decreased or replaced with the NLG1 SWAP mutant defective for TSPAN5 association failed to affect the proportion of mushroom spines. Interestingly, a significant effect was seen on dendritic spine density and in the proportion of thin and stubby spines, suggestive of other TSPAN5 functions (i.e., the interaction between TSPAN5 and ADAM-10) (Haining et al., 2012). Studying the quantitative relationship between other TSPAN5 interaction partners will be the focus of future work.

Finally, the effects on dendritic spine maturation caused by TSPAN5 knockdown can be rescued by forcing NLG1 clustering. This finding strengthens the hypothesis that TSPAN5 promotes NLG1 clustering and that this pathway is vital for dendritic spine maturation.

## STAR★Methods

### Key Resources Table

**Table:** 

REAGENT or RESOURCE	SOURCE	IDENTIFIER
**Antibodies**		

α-TSPAN5 Rabbit Polyclonal	Aviva System Biology	Cat# AV46640
α-Transferrin Receptor Mouse Monoclonal	Thermo Fisher	Clone H68.4
α-alpha Tubulin Mouse Monoclonal	Sigma Aldrich	Cat. No T5168
α-PSD95 Mouse Monoclonal	Neuromab	RRID AB_10698024
α-GluA2 Mouse Monoclonal	Neuromab	RRID AB_10674575
α-Bassoon Mouse Monoclonal	Neuromab	RRID AB_2716712
α-VGlut1 Mouse Monoclonal	Synaptic Systems	Cat. No. 135 311
α-VGAT Mouse Monoclonal	Synaptic Systems	Cat. No. 131 011
α-GABAAβ3	Neuromab	RRID AB_10673389
α-VGlut1 Rabbit Polyclonal	Synaptic Systems	Cat. No. 135 303
α-neuroligin-1 Rabbit Polyclonal	Synaptic Systems	Cat. No. 129 013
α-GFP Rabbit Polyclonal	MBL	Code No. 598
α-GFP Rabbit Polyclonal	Chromotek	Code PABG1
α-GFP Chicken	Aves	Cat. No. GFP-1010
α-CamKII alpha Goat Polyclonal	Abcam	Code No Ab87597
α-GluA2/3 Rabbit Polyclonal	Dr C. Gotti	N/A
α-GluA2 Mouse Monoclonal	Dr D. Choquet	N/A
α-HA Rat Monoclonal	Roche	Cat. No. 11867423001
α-pan Neurexin-1 Rabbit Polyclonal	Millipore	Cat. No. ABN161-I
α-neuroligin-3 Rabbit Polyclonal	Synaptic Systems	Cat. No. 129113
α-N-Cadherin Mouse Monoclonal	BD transduction lab	Cat. No. 610920
α-N-Cadherin Rabbit Polyclonal	Abcam	Code ab18203
α-giantin Mouse	Dr. H. P. Hauri	N/A
α-Rabbit IgG-Alexa 488	Invitrogen	Cat. No A11034
α-Rabbit IgG-Alexa 532	Jackson ImmunoResearch	N/A
α-Rabbit IgG-Alexa 555	Invitrogen	Cat. No A21429
α-Rabbit IgG-DyLight 649	Jackson ImmunoResearch	Cat. No 211-492-171
α-Mouse IgG-Alexa 488	Invitrogen	Cat. No A11029
α-Mouse IgG-Alexa 555	Invitrogen	Cat. No A21424
α-Goat IgG-Cy5	Jackson ImmunoResearch	Cat. No 705-175-147
α-Rat IgG-Alexa 568	Invitrogen	Cat. No. A11077
α-Rat IgG-Alexa 647	Invitrogen	Cat. No. A21247
α-Chicken IgG-Alexa 633	Invitrogen	Cat. No. A21103
α- Rabbit IgG-HRP	GE Healthcare	Cat. No NA934V
α- Mouse IgG-HRP	GE Healthcare	Cat. No NA931V
α-Rat IgG-HRP	Jackson Immunoresearch	Cat. No. 112-005-003
α-Chicken IgY-HRP	Jackson Immunoresearch	Cat. No. 703-035
Streptavidin, Alexa 488 Conjugate	Invitrogen	Cat. No S32354
Monomeric Streptavidin, Atto 594 Conjugate	Dr O. Thoumine	N/A
Monomeric Streptavidin, Atto 647 Conjugate	Dr O. Thoumine	N/A
Neurexin1β-Fc	Dr O. Thoumine	N/A
α-human IgG Fcγ Fragment Specific	Jackson ImmunoResearch	Code: 109-005-098
α-human IgG Fcγ Fragment Specific Alexa 647	Jackson ImmunoResearch	Code: 109-605-098

**Bacterial and Virus Strains**		

E. Coli Dh5α	Invitrogen	N/A
E. Coli BL21	Invitrogen	N/A

**Experimental Models: Cell Lines**		

HEK293 FT	Thermo Fisher Scientific	R70007
HeLa	Dr S. Colombo	N/A

**Experimental Models: Organisms/Strains**		

Wistar Rats	Charles River laboratories	N/A
C57BL/6	Charles River Laboratories	N/A
C57BL/6 TSPAN5 KO	Saint-Pol et al., 2017	N/A
CD1	Charles River Laboratories	N/A

**Recombinant DNA**		

pLVTHM-Scrambled	In House	N/A
pLVTHM-Sh-TSPAN5	In House	N/A
pSICOR-UBC-Sh-TSPAN5+ TSPAN5-GFP (Rescue)	In House modified from Addgene #11579; Ventura et al., 2004	N/A
pSICOR-UBC-ShTSPAN5 + TSPAN5-DLQ-GFP (DLQ)	In House modified from Addgene #11579; Ventura et al., 2004	N/A
pSICOR-UBC-ShTSPAN5 + TSPAN5-NIYF-GFP (NIYF)	In House modified from Addgene #11579; Ventura et al., 2004	N/A
pSICOR-UBC-ShTSPAN5 + TSPAN5-PLM-GFP (PLM)	In House modified from Addgene #11579; Ventura et al., 2004	N/A
pSICOR-Scrambled + mCherry	In House modified from Addgene #21907; Gaspar-Maia et al., 2009	N/A
pSICOR-ShTSPAN5 + mCherry	In House modified from Addgene #21907; Gaspar-Maia et al., 2009	N/A
pSICOR-ShTSPAN5 + TSPAN5-mCherry	In House modified from Addgene #21907; Gaspar-Maia et al., 2009	N/A
pSICOR-ShTSPAN5 + TSPAN5-DLQ-mCherry	In House modified from Addgene #21907; Gaspar-Maia et al., 2009	N/A
pSICOR-ShTSPAN5 + TSPAN5-NIYF-mCherry	In House modified from Addgene #21907; Gaspar-Maia et al., 2009	N/A
TSPAN5-GFP	Dr E. Rubinstein	N/A
TSPAN5-PLM	Dr E. Rubinstein	N/A
TSPAN5-RDD	Dr E. Rubinstein	N/A
TSPAN5-DID	Dr E. Rubinstein	N/A
TSPAN5-DLQ	Dr E. Rubinstein	N/A
TSPAN5-NIYF	Dr E. Rubinstein	N/A
CD9-GFP	Dr E. Rubinstein	N/A
CD81-GFP	Dr E. Rubinstein	N/A
pGEX4Ti1	In House	N/A
pGEX4Ti1-Ct	In House	N/A
pGEX4Ti1-LEL	In House	N/A
pGEX4Ti1-LEL-RDD	In House	N/A
pGEX4Ti1-LEL-DID	In House	N/A
pGEX4Ti1-LEL-DLQ	In House	N/A
pGEX4Ti1-LEL-NIYF	In House	N/A
AP-neuroligin-1	Dr Alice Ting	N/A
BirA-ER	Dr Alice Ting	N/A
HA-neuroligin-1	Dr. O. Thoumine	N/A
HA-neuroligin-1-SWAP	Dr. O. Thoumine	N/A
HA-neuroligin-1-ΔC	Dr. O. Thoumine	N/A
HA-neuroligin-3	Dr. O. Thoumine	N/A
Sh-neuroligin-1	Dr. O. Thoumine	N/A
mEOS-N-Cadherin	Dr. O. Thoumine	N/A
SEP-N-Cadherin	Dr. Xiang Yu	N/A

**Software and algorithms**		

NeuronStudio 0.9.92	Open source software – developed by S.L. Wearne and P.R. Hof, Computational Neurobiology and Imaging Center, Mount Sinai School of Medicine, New York, NY	N/A
BioRender	BioRender.com	N/A
Clampex 10.1	Axon Instruments, Molecular Devices	N/A
Clampfit 10.1	Axon Instruments, Molecular Devices	N/A
Metamorph macro based for tracking of uPAINT experiments	Izeddin et al., 2012, Kechkar et al., 2013	N/A
MetaMorph plug-in for analysis of SptPALM experiments	Chamma et al., 2016a	N/A

### Lead Contact and Materials Availability

Further information and requests for resources and reagents should be directed to and will be fulfilled by the Lead Contact, Maria Passafaro (maria.passafaro@in.cnr.it).

Plasmids generated in this study are available upon request with Material Transfer Agreements.

### Experimental Model and Subject Details

Animal procedures were performed in accordance with the European Community Council Directive of November 24, 1986 (86/609/EEC) on the care and use of animals and following the guidelines of the UCL-Institute of Neurology Genetic Manipulation and Ethic Committees under license from the UK Home Office in accordance with the Animals (Scientific Procedures) Act 1986 (Amended Regulations 2012). Animal procedures were approved by the Italian Ministry of Health (Protocol Number N° 100/2016) and by the France Ministry of Agriculture (N° 742/2014) for primary cultures from rat embryos and for In utero electroporation (Iacuc 799).

HEK293 or HeLa (Thermo Fisher Scientific) cells were grown in DMEM (GIBCO), supplemented with 10% FBS (GIBCO), 1% L-glutamine (Invitrogen), 0.1% gentamycin (Invitrogen) incubated at 37 °C with 5% CO_2_. Transfected cells used for streptavidin pulldown experiments were grown for 48 h in Neurobasal (GIBCO) medium supplemented with 2% B27 (prepared as in Chen et al. 2008), 0.25% L-glutamine and 1% penicillin/streptamycin (Invitrogen).

The 293FT cell line used to generate the lentiviruses was grown in DMEM supplemented with 10% FBS, 1% L-glutamine, 0.1% gentamycin and 0.1% G418 (Invitrogen).

Primary hippocampal neurons were prepared from either Wistar E18 rat brains (Folci et al., 2014, Valnegri et al., 2011, Zapata et al., 2017) or C57BL/6 P0 mice. Neurons were plated onto coverslips coated overnight with 0.25 mg ml^−1^ poly-D-lysine (Sigma Aldrich) at 75,000 per well and grown in Neurobasal medium supplemented with 2% B27 (prepared as in Chen et al. 2008), 0.25% L-glutamine, 1% penicillin/streptomycin and 0.125% Glutamate (Sigma Aldrich).

The mice used for in utero electroporation were pregnant CD1 outbred and embryos were electroporated at E13.5. Positive male animals were perfused at P30.

Adult rat used for hippocampus and cortex lysates were 3-month old male Wistar.

### Method Details

#### Plasmids

pLVTHM-ShTSPAN5 was obtained by ligation of previously described ShRNA sequence specific for rat TSPAN5 (CAGGACAATTTAACCATTGTG) (Dunn et al., 2010) into pLVTHM vector, at MluI/ClaI sites. pLVTHM-Scrambled was obtained by inserting a sequence derived by random mixing of the bases from ShTSPAN5 (GCAAATTCGTGTCGTATAACA) in pLVTHM sites MluI/ClaI.

pSICOR was obtained from Addgene #11579 and modified by substitution of the CMV promoter with a human ubiquitin C (UBC) promoter in restriction sites NotI/NheI. The Sh-TSPAN5 sequence was inserted at the HpaI/XhoI sites and human TSPAN5 cDNA was subcloned from TSPAN5-GFP (Dr Rubinstein) and inserted upstream of the EGFP sequence at sites NheI/AgeI. Please note that the human TSPAN5 nucleotidic sequence differs from that of rat in the site of the ShRNA thus allowing to use it as shRNA-resistant sequence.

The same procedure was used to produce the DLQ, NIYF and PLM pSICOR constructs by subcloning from TSPAN5-DLQ-GFP, TSPAN5-NIYF-GFP and TSPAN5-PLM-GFP (Saint-Pol et al., 2017).

pSICOR-mCherry was obtained from Addgene #21907 and Scrambled or ShRNA sequences were inserted in the HpaI/XhoI sites and the cDNA of human wild-type TSPAN5, DLQ and NIYF mutants were inserted in NheI/AgeI sites.

TSPAN5-RFP was produced by PCR and inserted in tDimer-RFP vector in the EcoRI site.

pGEX-4T-1 constructs were produced by PCR from TSPAN5-GFP, TSPAN5-RDD-GFP, TSPAN5-DID-GFP, TSPAN5-DLQ-GFP and TSPAN5-NIYF-GFP (Dr Rubinstein) and by insertion at the BamHI/EcoRI sites.

#### Transfection and Infection

HEK293 cells were transfected with the calcium phosphate method. Briefly, DNA (2 μg x 6-well) was mixed with 130mM CaCl_2_ in H_2_0 (200 μl per well). One volume of HEBS buffer (280 mM NaCl, 100 mM HEPES, 1.5 mM Na2HPO4, pH 7.11) was added to the DNA and thoroughly mixed to produce air bubbles. The mix was added to the cells and left for 5 h before washing and changing the medium.

DIV5 rat or mouse hippocampal neurons were transfected with Lipofectamine 2000 (Invitrogen) or infected with lentiviral particles produced as previously described (Lois et al., 2002).

#### BS3 crosslinking

The experiments were carried out according to Boudreau et al. (2012). Briefly, primary hippocampal neurons were washed twice with PBS supplemented with 0.1mM CaCl_2_ (Sigma Aldrich) and 1 mM MgCl_2_ (Sigma Aldrich) at 37°C. Neurons were then exposed to PBS supplemented with 0.1mM CaCl_2_ and 1 mM MgCl_2_ with and without BS3 crosslinker (1mg/ml, ThermoFisher) at 4°C for 10 min. Neurons were then rapidly washed first with TBS supplemented with 0.1 mM CaCl_2_ and 1 mM MgCl_2_ plus 50 mM glycine (Sigma Aldrich) at 4°C and then with TBS supplemented with 0.1 mM CaCl_2_ and 1 mM MgCl_2_ at 4°C prior to lysis with BS3 buffer (50 mM Tris-HCl, 150 mM NaCl, 1 mM EDTA, pH 7.4, 1% SDS plus protease inhibitors). 3X Laemmli sample buffer was then added and the samples analyzed by SDS-PAGE and western blotting.

#### Synaptosomes purification

Hippocampi and cortices were collected from adult Wistar rats and homogenized with glass-teflon potter in homogenization buffer (0.32 M sucrose, 10 mM HEPES-NaOH, protease inhibitors, pH 7.4). The total homogenate was centrifuged at 1,000 g for 10 min at 4°C. The pellet P1 corresponds to the nuclear fraction. The supernatant S1 was centrifuged at 10,000 g for 15 min at 4°C. The resulting pellet (P2) corresponds to crude synaptosomal fraction while the supernatant (S2) contains cytosolic components and light membranes. The P2 fraction was resuspended in homogenization buffer and centrifuged again at 10,000 g for 15 min at 4°C to wash the synaptosomes. Crude synaptosomes were loaded on top of a discontinuous sucrose gradient (0.8, 1, 1.2 M) and centrifuged at 150,000 g at 4°C for 2h. Purified synaptosomes were collected between 1 and 1.2 M sucrose layers. The fractions were all resuspended in homogenization buffer. 3X sample buffer was then added and the samples analyzed by SDS-PAGE and western blot by loading the same amount of proteins.

#### Immunoprecipitation, Streptavidin-precipitation or GFP-Trap precipitation

For immunoprecipitation experiments, hippocampi and cortices were dissected from adult rat brain, pooled together and lysed in RIPA buffer (50 mM Tris, 150 mM NaCl, 1 mM EDTA, 1% NP40, 1% Triton X-100, pH 7.4, protease inhibitor) in teflon-glass potter, rotated for 1h at 4°C and centrifuged at 10,000 g for 30 min at 4 °C. Supernatants were incubated with antibodies at 4 °C overnight. Protein A-agarose beads (GE Healthcare, USA) were incubated with the supernatants at 4 °C for 2 h. Beads were washed three times with RIPA buffer, resuspended in 3X sample buffer and analyzed by SDS–PAGE followed by western blotting.

For streptavidin-precipitation experiments, transfected HEK293 cells were lysed in RIPA buffer (50 mM Tris-HCl, 150 mM NaCl, 1 mM EDTA, 1% NP40, 1% Triton X-100, pH 7.4, protease inhibitor), rotated for 1h at 4°C and centrifuged at 10,000 g for 30 min at 4 °C. Supernatants were incubated with streptavidin immobilized on agarose beads (Thermo Scientific) for 3 h at 4°C. The beads were first precipitated by centrifugation at 4,000 RPM for 5 min at 4°C, washed three times with RIPA buffer and then resuspended in 3X sample buffer and analyzed by SDS–PAGE followed by western blotting.

For GFP-Trap precipitation, transfected HEK293 cells were lysed in RIPA buffer (50 mM Tris, 150 mM NaCl, 1 mM EDTA, 1% NP40, 1% Triton X-100, pH 7.4, protease inhibitor), rotated for 1 h at 4°C and centrifuged at 10,000 g for 30 min at 4 °C. The supernatants were first incubated with GFP-Trap magnetic beads (ChromoTek) for 1 h at 4°C and then precipitated by magnetic separation and washed three times with RIPA buffer, resuspended in 3X sample buffer and analyzed by SDS–PAGE followed by western blotting.

#### GST pulldown

GST-fusion proteins were prepared by growing transformed BL21 *E. coli* and inducing recombinant protein expression by adding IPTG (0.5mM final concentration) for 2 h. Bacteria were pelleted, resuspended in lysis buffer (8 M urea, 50 mM Tris-HCl, 1 mM EDTA, 1 mM DTT, pH 7.5) and rotated for 1 h at 4°C. Lysed bacteria were inserted into a dialysis membrane (cutoff 13kDa) and dialyzed for 1 h at 4°C in dialysis buffer I (4 M urea, 1 mM Tris-HCl, 1 mM DTT, pH 5). The membrane was then moved to dialysis buffer II (50 mM Tris-HCl, 50 mM NaCl, 2 mM MgCl2, 200 μM DTT, pH7.5) overnight at 4°C. Membranes were dialyzed with new dialysis buffer II for 3 h at 4°C. Lysates were then collected and centrifuged at 10,000 g for 30 min at 4°C. The supernatant was incubated with glutathione Sepharose beads (Thermo Scientific) overnight at 4°C and then washed with dialysis buffer II.

Hippocampi and cortices dissected from adult rat brains were pooled together, lysed in RIPA buffer by homogenization in a teflon-glass potter, rotated for 1 h at 4°C and then centrifuged at 10,000 g for 30 min at 4 °C. Supernatants were incubated with glutathione Sepharose beads for 3 h at 4°C and then washed and resuspended in 3X sample buffer and analyzed by SDS-PAGE followed by western blotting.

#### Western blots

Proteins were transferred from the acrylamide gel onto the nitrocellulose (0.22 μm, GE Healthcare) or PVDF membranes. Membranes were incubated with the primary antibodies (α-TSPAN5 1:500; α-transferrin receptor 1:500; α-tubulin 1:40,000; α-PSD-95 1:1,000; α-synaptophysin 1:500; α-NLG1 1:500; α-GluA2 1:500; α-GluA2/3 1:2,000; α-N-Cadherin 1:500; α-neuroligin-3 1:1,000; α-GFP 1:2,500; α-HA 1:1,000, see Key Resource Table for Catalogue Numbers) at room temperature for 2–3 h or overnight at 4°C in TBS Tween-20 (0.1%), milk (5%). After washing, the blots were incubated at room temperature for 1 h with horseradish peroxidase-conjugated α-rabbit, α-mouse or α-rat antibodies (1:2,000) in TBS Tween-20 (0.1%), milk (5%). Immunoreactive bands on blots were visualized by enhanced chemiluminescence (GE Healthcare).

#### Real-time PCR

Mouse hippocampal cultures were prepared as reported previously (Beaudoin et al., 2012). To modulate the expression of TSPAN5, mouse hippocampal neurons were infected at DIV5 with lentiviral particles carrying either Scrambled, Sh-TSPAN5 or Rescue DNA. mRNA was extracted using Nucleozol Reagent following manufacturer instructions (Macherey Nagel) from neurons at DIV12.

For each condition, 1.5 ug of extracted mRNA was used to synthetize cDNA using SuperScript VILO cDNA Synthesis Kit (Thermo Fisher).

The target sequences of TSPAN5, TSPAN7, CD81 and α-actin (endogenous control) were amplified from 60 ng of cDNA in the presence of SYBR Green PCR Master Mix (Applied Biosystems) using Applied Biosystems 7000 Real-Time thermocycler. Primer sequences were as follows: mouse TSPAN5 Fw (AACAACATCAGAGCCTACAGAG), mouse TSPAN5 Rev (GGTTCCAATCATCAGCTCCA), TSPAN7 Fw (ACCAGTTTTATGGAGACTAACATGG), TSPAN7 Rv (AGCAGCATGCCAATCAACT), CD81 Fw (TGATGATGTTTGTAGGCTTCCT), CD81 Rv (CTCACAGGCAAACAGGATCA), α-actin Fw (AGATGACCCAGATCATGTTTGAGA), α-actin Rev (CCTCGTAGATGGGCACAGTGT)

Each sample was run in triplicate, and the results were calculated using the ΔΔCT method to allow the normalization of each sample to the internal standard and comparison with the calibrator of each experiment.

#### Electrophysiology

Miniature excitatory or inhibitory post-synaptic currents (mIPSCs/mEPSCs) were recorded in the presence of the voltage-dependent sodium channels blocker (500 μM lidocaine). Additional blockers were added including the broad-spectrum glutamatergic blocker kynurenic acid (3 mM) and the GABA_A_Rs blocker bicuculline (20 μM) to isolate mIPSCs and mEPSCs respectively. The composition of the intracellular solution was 126 mM K-gluconate, 4 mM NaCl, 1 mM EGTA, 1 mM MgSO_4_, 0.5 mM CaCl_2_, 3 mM ATP (magnesium salt), 0.1 mM GTP (sodium salt), 10 mM glucose, 10 mM HEPES-KOH (pH 7.3; osmolarity adjusted to 280 mOsm). Mixed AMPA/NMDA-mEPSCs were recorded in absence of Mg^2+^ to reduce Mg^2+^ block of NMDARs. Pure AMPAR mediated currents were isolated by perfusing neurons for at least 6 minutes with an NMDAR blocker (100 μM APV) to estimate the time when the AMPAR component of mixed AMPA/NMDA-mEPSCs decays. This allowed the measure of the NMDAR component of mixed AMPA/NMDA-mEPSCs. Specifically, the AMPAR component of mixed events were measured at the peak of the current while the NMDAR component were measured in a window between 10-20 ms after the AMPAR peak (adapted from Watt et al., 2000 and Myme et al., 2003). The internal solution used for recordings was the same as for mIPSCs or mEPSCs but supplemented with lidocaine *N*-ethyl bromide (QX-314 5 mM).

The readily releasable pool (RRP) size was evaluated by perfusing neurons for 5 s with KRH supplemented with 1 M sucrose as previously described (Antonucci et al., 2013).

Recordings were performed with a Multiclamp 700B amplifier (Axon CNS molecular devices, USA). Pipette resistance was 2–3 MΩ and series resistance always below 20 MΩ. mEPSCs and RRP size were recorded at a holding potential of −70 mV, filtered at 2 kHz, and digitized at 20 kHz using Clampex 10.1 software (Axon Instruments, Molecular Devices). Analysis was performed offline with Clampfit 10.1 software (Axon Instruments, Molecular Devices) using a threshold crossing principle. Cells with noisy or unstable baselines were discarded.

#### Immunocytochemistry

Cultured hippocampal neurons were washed in PBS supplemented with 0.1 mM CaCl_2_ and 1 mM MgCl_2_ and fixed in paraformaldehyde (4%, Sigma Aldrich)/sucrose (4%, Sigma Aldrich) for 10 min at room temperature or in methanol (Sigma Aldrich) for 10 min at 4°C and incubated with primary antibodies (α-TSPAN5 1:50; α-PSD-95 1:200; α-GluA2: 1:200; α-Bassoon 1:500; α-VGlut1 1:200; α-VGAT 1:200; α-GABAAβ3 1:200, see Key Resource Table for Catalogue Numbers) in GDB1X solution (2X: 0.2% gelatin, 0.6% Triton X-100, 33mM Na_2_HPO_4_, 0.9 M NaCl, pH 7.4) for 2 h at room temperature.

For neurexin staining neurons were washed in PBS supplemented with 0.1 mM CaCl_2_ and 1 mM MgCl_2_ and fixed in paraformaldehyde (4%, Sigma Aldrich)/ sucrose (4%, Sigma Aldrich) for 10 min at room temperature and then blocked for 1 h with blocking solution (10% goat serum, 0.5% BSA in PBS) followed by 3 h incubation with α-pan neurexin-1 (1:250) in primary antibody solution (10% goat serum, 0.5% BSA, 0.2% Triton X-100 in PBS).

For surface staining, antibodies (streptavidin-488 1:100; α-HA 1:15; α-GFP 1:500) were applied to neurons for 10 min at room temperature followed by a washing step in PBS supplemented with 0.1 mM CaCl_2_ and 1 mM MgCl_2_ and paraformaldehyde fixation.

After three washes with high salt buffer (500mM NaCl, 20mM NaPO_4_^2-^, pH 7.4) the coverslips were incubated with secondary antibodies (Alexa-conjugated: 1:400; DyLight-conjugated: 1:300) in GDB1X solution for 1h at room temperature.

Coverslips were washed with high salt buffer and mounted with Mowiol (Sigma Aldrich).

Fluorescence images were acquired with an LSM510 Meta confocal microscope (Carl Zeiss; gift from F. Monzino) and a 63X oil-immersion objective (numerical aperture 1.4) with sequential acquisition setting, at 1,024 X 1,024 pixels resolution. Images were Z series projections of approximately 6–10 images, each averaged four times and taken at depth intervals of 0.75 μm.

Dendritic spines were counted on all GFP positive neuronal dendritic arbor excluding the soma and classified with NeuronStudio software (NeuronStudio©) according to the following parameters: General parameters for spine identification: Length > 0.2 μm and < 3.0 μm, Max Width 3.0 μm, Stubby spines size > 10 voxels, Non Stubby spines size > 5 voxels. For spine types classifications the following logical tests were used: if Neck Ratio (head/neck diameter) > 1.100 then a spine was classified as Thin (if also spine length/head diameter > 2.5) or Mushroom (if also head diameter was > 0.35 μm). A spine is classified as Stubby if it fails at any of the precedent logical tests.

Sholl analysis was performed with the NeuronStudio software using concentric circles with 3 μm radius.

Puncta or clusters were calculated with Fiji software thresholding images at intensity equal to 3 X StDev of the signals.

For Figure 1C only clusters with more than 50% overlap were considered as colocalizing.

For Figure 3A, only GFP positive areas were considered for the analysis and we did not apply any threshold to the % of overlap.

#### NLG1 clustering experiment

NLG1 clustering was induced by application of clustered neurexin1β-Fc. Purified neurexin1β -Fc (Mondin et al., 2013) were pre-clustered by mixing it with an anti-human-Fc (in a 2:1 weight ratio) for 5 min at 37°C in Neurobasal medium.

Non-clustered or clustered neurexin1β -Fc were applied to DIV12 neurons in media and left for two days.

α-HA and α-human Fc-Alexa647 were applied for 10 min at room temperature in PBS supplemented with 0.1 mM CaCl_2_ and 1 mM MgCl_2_ to surface stain both HA-tagged-NLG1 and neurexin1β-Fc.

Neurons were then fixed with paraformaldehyde (4%)/sucrose (4%) for 10 min at room temperature, washed and stained with a secondary-Alexa568 antibody to detect α-HA signal.

After imaging as above, both the Alexa568 and Alexa647 signals were thresholded for intensity equal to three times the StDev of the signals. Only puncta positive for both channels were considered in the analysis.

#### In utero electroporation and brains processing for imaging

All procedures were approved by the Italian Ministry of Health and the San Raffaele Scientific Institute Animal Care and Use Committee in accordance with the relevant guidelines and regulations. Electroporation in utero was employed to deliver the expression vectors to the ventricular RGCs of CD1 mouse embryos as previously described (Saito, 2006). Briefly, uterine horns of E13.5 pregnant dams were exposed by midline laparotomy after anesthesia with Avertin (312 mg/kg). 1 μl of DNA plasmid corresponding to 3 μg mixed with 0.03% fast-green dye in PBS was injected in the telencephalic vesicle using a pulled micropipette through the uterine wall and amniotic sac. 7 mm platinum tweezer-style electrodes were placed outside the uterus over the telencephalon and 5 pulses of 40 V, of 50 ms duration, were applied at 950 ms intervals by using a BTX square wave electroporator. The uterus was then placed back in the abdomen, the cavity was filled with warm sterile PBS and the abdominal muscle and skin incisions were closed with silk sutures.

Animals with GFP and tdTomato positive signals in the brain were aged to P30 and then anesthetized with 10 mg/ml Avertin and intracardially perfused with 4% paraformaldehyde/ 4% sucrose. Brains were collected, 150 μm thick slices were cut with a vibratome (Leica) and mounted on polylysine-covered coverglass (VWR) with FluoroMount (Sigma Aldrich).

Secondary dendrites of around 60μm from GFP and tdTomato positive pyramidal cortical neurons were imaged with a Z-projection of 0.35 μm steps with the confocal ZEISS LSM 800 microscope mounting a 63X oil immersion objective at 1024X1024 pixels resolution. Dendritic spines were counted manually from secondary branches of the apical dendrite of layer III pyramidal neurons in the lateral somatosensory cortex.

#### uPAINT

Transfected rat hippocampal cultured neurons were imaged either with monomeric streptavidin coupled to Atto-594 (Chamma et al., 2016a, Chamma et al., 2016b, Chamma et al., 2017) to image overexpressed AP-NLG1 mobility or with α-GluA2 antibody coupled to Atto-657 to study GluA2 AMPARs mobility (Giannone et al., 2010, Giannone et al., 2013, Nair et al., 2013).

Briefly, coverslips were mounted in an open Inox chamber (Life imaging services) and the growth medium was replaced by Tyrode solution (15 mM D-glucose, 108 mM NaCl, 5 mM KCl, 2 mM MgCl2, 2 mM CaCl2 and 25 mM HEPES-NaOH, pH 7.4) containing 1% globulin-free BSA (Sigma). A Nikon Ti-E Eclipse inverted microscope equipped with an EMCCD camera (Evolve, Roper Scientific, Evry, France) and an apochromatic (APO) total internal reflection fluorescence (TIRF) 100X/1.49 numerical aperture (NA) oil objective was used for imaging. GFP positive neurons were selected and low concentrations of Atto 594-conjugated mSA (1 nM) or Atto 647- conjugated α-GluA2 antibody were added to isolate single molecules. A four-color laser bench (405; 488; 561; and 642 nm, 100 mW each; Roper Scientific) was connected through an optical fiber to the TIRF illumination arm of the microscope. Laser powers were controlled through acousto-optical tunable filters driven by the Metamorph software (Molecular Devices, USA). Atto 594 and Atto 647 were excited with the 561- and 642-nm laser lines through a four-band beam splitter (BS R405/488/561/635, SemRock). Samples were imaged by oblique laser illumination, allowing the excitation of individual Atto-conjugated ligands bound to the cell surface, without illuminating ligands in solution. Atto 594 and 647 fluorescence was collected using FF01-617/73 and FF01-676/29 nm emission filters (SemRock), respectively, placed on a filter wheel (Suter). Stacks of 4,000 consecutive frames were obtained from each cell, with an integration time of 20 ms. Acquisitions were steered using the Metamorph software (Molecular Devices) in a streaming mode at 50 Hz.

Images were analyzed with a custom Metamorph macro based on wavelet segmentation for localization and simulated annealing algorithms for tracking, previously described (Izeddin et al., 2012, Kechkar et al., 2013). Super-resolution images (Detections) were built by summing the localizations of all detected single molecules across the stack. Tracks of localized molecules were calculated by following the position of single molecules through successive frames. The diffusion coefficient, D, was calculated for each trajectory from linear fits of the first 4 points of the mean square displacement (MSD) function versus time.

#### mEOS tracking

mEOS-tagged N-Cadherin was generated as previously described (Garcia et al., 2015). Hippocampal neurons were transfected at DIV 7 using Effectene (QIAGEN) according to the manufacturer instructions. Spt-PALM experiments were performed at DIV 12. Coverslips were mounted in an open Inox chamber (Life Imaging Services) placed on the 2D stage of a fully motorized epifluorescence microscope (Nikon Eclipse TiE), equipped with a 100 × /1.49-N.A. objective. A four-color laser bench (405 nm, 488 nm, 561 nm, and 642 nm; 100 mW each; Roper Scientific) was connected to the TIRF illumination arm of the microscope through an optical fiber. Laser power was controlled through an acousto-optical tunable filter driven by MetaMorph software (Molecular Devices). Filter sets were from SemROCK: EGFP (excitation: FF01-472/30, dichroic: FF-495Di02, emission: FF01-520/35) and mEos2 (excitation: laser 405/561 nm, dichroic: Di01-R561, emission: FF01-617/73). Cells expressing green-emitting mEos2-tagged molecules and/or EGFP were imaged using the EGFP filter, and single photoconverted mEos2 molecules (red-emitting) were imaged using the mEos2 filter upon simultaneous illumination with the 561-nm excitation laser (∼5 mW at the back aperture of the objective) and the 405-nm photoconversion laser (∼0.5 mW). This illumination mode allowed clear identification and localization of a subset of individual molecules over time. Illumination was performed in TIRF mode, allowing visualization of molecules at the surface membrane of dendrites. Images were captured using an EMCCD camera (Evolve; Photometrics). Sequences of 4,000 images were acquired in streaming mode at 50 Hz (20-ms exposure time) to capture fast-diffusing membrane-associated molecules. SptPALM experiments were analyzed using a custom-made algorithm written as a MetaMorph plug-in as previously described (Chamma et al., 2016a).

#### dSTORM

Primary cultured neurons were surface-labeled with 100 nM of mStrav-Alexa647 in Tyrode solution for 10 min, rinsed, and fixed with pure methanol 8 min at −20°C. Neurons were permeabilized using 0.2% Triton X-100 (Sigma) for 5 min, rinsed, blocked using 0.1% BSA (Sigma) in PBS for one hour, then immunolabeled for TSPAN5 (1:50), and incubated with Alexa 532 conjugated anti-rabbit secondary antibody (Jackson Immunoresearch). dSTORM imaging of cultured neurons was performed using an inverted motorized microscope (Nikon Ti, Japan) equipped with a x100 1.49NA PL-APO objective and a perfect focus system, allowing long acquisition in oblique illumination mode, 1W 532 and 647 laser lines. Both the ensemble and single-molecule fluorescence were collected by using a quad-band dichroic filter (Di01-R405/488/561/635, Semrock). The fluorescence was collected using a sensitive EMCCD (Evolve, Photometrics, USA). 100-nm fluorescent beads (Tetraspeck, Life Technologies) adhered to the surface of the coverslips were used as registration markers. Single-molecule localization and reconstruction was performed with automatic feedback control of the lasers using WaveTracer module, enabling optimal single-molecule density during the acquisition (Kechkar et al., 2013). The acquisition and localization sequences were driven by MetaMorph (Molecular Devices) in a streaming mode at 50 frames per second (20-ms exposure time) using an area equal to or less than 256 × 256 pixel region of interest, sequentially. Image registration and color alignment was performed using a custom registration program running on MetaMorph. The number of clusters was determined by wavelet segmentation based on areas with strong signal intensity compared to neighboring areas (Izeddin et al., 2012, Kechkar et al., 2013) on the super-resolved dSTORM images generated from 20,000 – 40 000 frames for TSPAN5 and NLG1 signal. Segmented images were then overlapped and the % of TSPAN5 clusters containing NLG1 cluster in synaptic areas (identified by morphology of the GFP signal) was quantified, as well as the area of overlap when colocalization was observed. No threshold of % of overlapping was considered.

#### Graphical Abstract

The graphical abstract was prepared using the BioRender software (Biorender.com).

### Quantification and Statistical Analysis

All statistical analyses were done with GraphPad Prism 6 software.

Two-tailed unpaired t test was performed to assess statistical significance between two independent groups (Figures S1D, S1I, S2A, S2B, and S4A). One-way ANOVA followed by Newman-Kuls post hoc multiple comparison test was used to assess statistical significance between three or more groups (Figures S1F, S1G, 2B, 2D, 2F, 3B, 3D, 5A–5D, 6B, 6D, 6F, S2C–S2D, S4B, S4C, S5A, S5B, S6A, S6B, and S7C–S7E).

Statistical details of the experiments can be found in the Table S1 and in figure legends (exact mean values, standard errors of the mean (SEM) and n).

Western blots were repeated at least three times from three independent experiments. Imaging experiments on cultured neurons were done on at least three independent cultures.

### Data and Code Availability

This study did not generate any datasets or code.

## Supplemental Information

Document S1. Figures S1–S7 and Table S1

Document S2. Article plus Supplemental Information
