# Supplementary material for: TSPAN5 Enriched Microdomains Provide a Platform for Dendritic Spine Maturation through Neuroligin-1 Clustering
Source: Cell Rep. 2019 Oct 30;29(5):1130–1146.e8. doi: 10.1016/j.celrep.2019.09.051 (PMC6899445; doi:10.1016/j.celrep.2019.09.051)
Supplement: Document S1. Figures S1–S7 and Table S1 [file mmc1.pdf]

Cell Reports, Volume *29*

Supplemental Information

TSPAN5 Enriched Microdomains Provide a Platform for Dendritic Spine Maturation through Neuroligin-1 Clustering

Edoardo Moretto, Anna Longatti, Luca Murru, Ingrid Chamma, Alessandro Sessa, Jonathan Zapata, Eric Hosy, Matthieu Sainlos, Julien Saint-Pol, Eric Rubinstein, Daniel Choquet, Vania Broccoli, Giampietro Schiavo, Olivier Thoumine, and Maria Passafaro

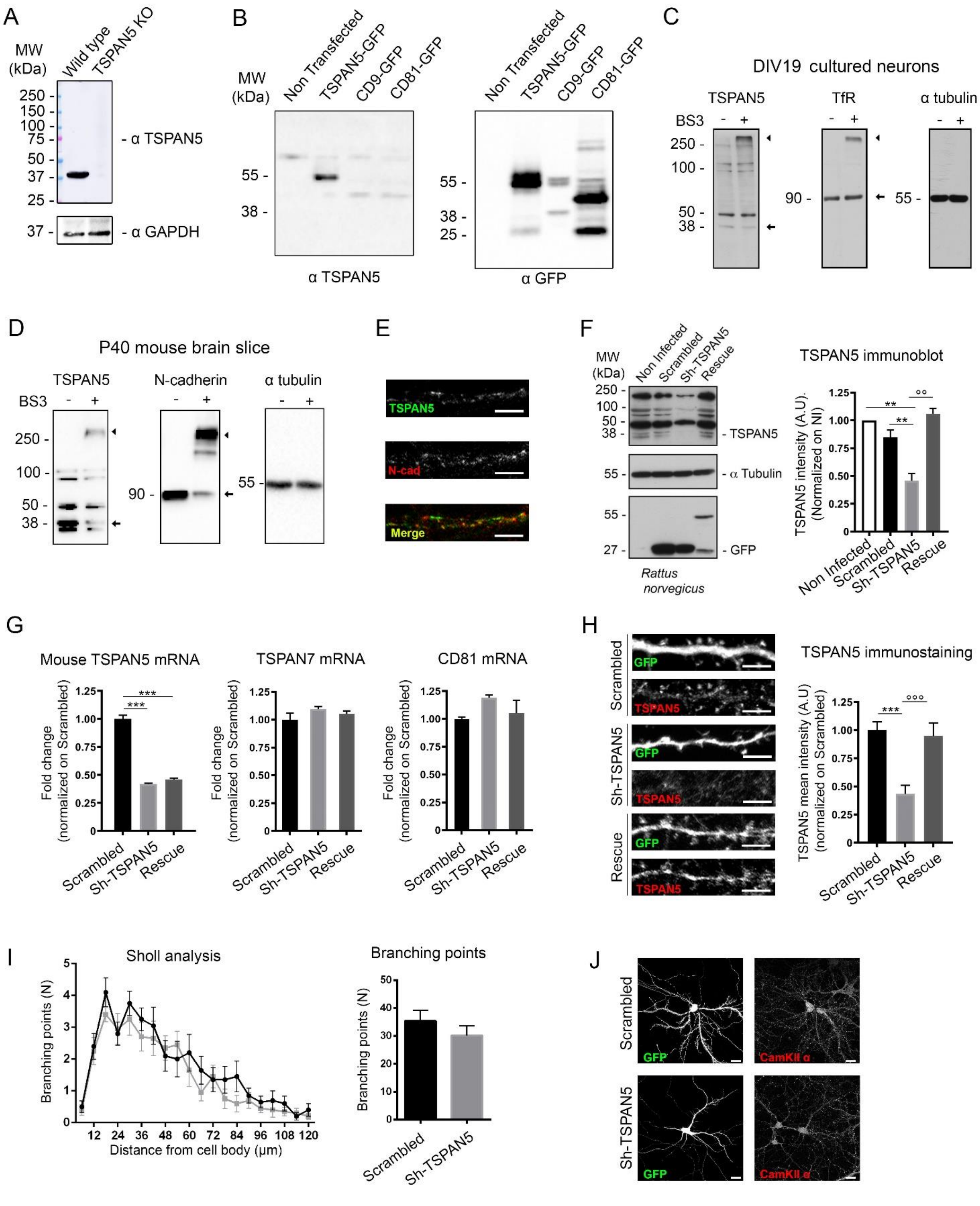

A
MW (kDa)
Wild type
TSPAN5 KO
250
150
100
75
50
37
25
- α TSPAN5
37
- α GAPDH
B
MW (kDa)
Non Transfected
TSPAN5-GFP
CD9-GFP
CD81-GFP
55
38
α TSPAN5
55
38
25
α GFP
C
DIV19 cultured neurons
TSPAN5
TfR
α tubulin
BS3
250
100
50
38
90
55
D
P40 mouse brain slice
TSPAN5
N-cadherin
α tubulin
BS3
250
100
50
38
90
55
E
TSPAN5
N-cad
Merge
F
MW (kDa)
Non Infected
Scrambled
Sh-TSPAN5
Rescue
250
100
50
38
- TSPAN5
55
- α Tubulin
55
27
- GFP
Rattus norvegicus
TSPAN5 immunoblot
TSPAN5 intensity (A.U). (Normalized on NI)
G
Mouse TSPAN5 mRNA
TSPAN7 mRNA
CD81 mRNA
Fold change (normalized on Scrambled)
Scrambled
Sh-TSPAN5
Rescue
H
Scrambled
Sh-TSPAN5
Rescue
GFP
TSPAN5
TSPAN5 immunostaining
TSPAN5 mean intensity (A.U) (normalized on Scrambled)
I
Sholl analysis
Branching points (N)
Distance from cell body (µm)
Branching points
Scrambled
Sh-TSPAN5
J
Scrambled
Sh-TSPAN5
GFP
CamKII α

Fig S1

Fig S1. Related to Figure 1 and 2

A) Western blot showing α-TSPAN5 antibody immunoreactive bands in RIPA lysates from wild type or TSPAN5 KO mice using the α-TSPAN5 antibody used throughout the manuscript. α GAPDH is used as loading control.

B) Western blot showing α-TSPAN5 antibody immunoreactive bands in RIPA lysates of HeLa cells non-transfected or transfected with either TSPAN5-GFP, CD9-GFP or CD81-GFP. GFP was used as transfection control.

C) Western blot showing BS3 crosslinking experiments on DIV19 rat cultured neurons. Upon application of the BS3 crosslinker, there is the appearance of a band at higher molecular weight. TfR was used as surface protein controls in addition to Tubulin as loading control. N = DIV19 cultured neurons: 4 independent preparations.

D) Western blot showing BS3 crosslinking experiments on P40 mouse brain slices. Upon application of the BS3 crosslinker, there is the appearance of a band at higher molecular weight. N-cadherin was used as surface protein controls in addition to Tubulin as loading control. N = 3 animals

E) Confocal images showing DIV12 cultured hippocampal neurons immunolabelled for TSPAN5 (in green) and N-Cadherin (in red) to show colocalization in early post-synaptic formation. % of TSPAN5 puncta colocalizing with: N-cadherin: 58.92±4.00. Scale bar = 5 µm.

F) Left panel: Western blot showing TSPAN5 immunoreactive band in BS3 buffer lysates from DIV14 rat hippocampal cultured neurons either non-infected or infected at DIV5 with lentiviral particles carrying either Scrambled, Sh-TSPAN5 or Rescue DNA. There is a visible reduction of intensity of all the bands detected by the α-TSPAN5 antibody. Tubulin is used as loading control and GFP as infection control. Note that the Rescue construct produces both GFP fused TSPAN5 (around 60KDa) and soluble GFP. Right panel: quantification of the intensity of all the bands detected by the α-TSPAN5 antibody (TSPAN5 intensity (A.U.) (normalized on non-infected): Non-Infected 1; Scrambled 0.85±0.06; Sh-TSPAN5 0.46±0.09; Rescue 1.06±0.05).

G) Quantification of the semi-quantitative Realtime PCR with probes for mouse TSPAN5, TSPAN7 and CD81 transcripts (DDCt method normalized to beta Actin) from RNA extracted at DIV12 from mouse cortical neurons infected at DIV5 with lentiviral particles carrying Scrambled, Sh-TSPAN5 or Rescue DNA. A significant reduction of the mRNA occurs in both the Sh-TSPAN5 and Rescue condition (where the Sh-TSPAN5 resistant human TSPAN5 cDNA is expressed), whereas no changes are detected for other members of the tetraspanin superfamily TSPAN7 and CD81 (fold change (normalized to Scrambled): TSPAN5: Scrambled 1.00±0.03; Sh-TSPAN5 0.42±0.01; Rescue 0.46±0.01; TSPAN7: Scrambled 1.00±0.06; Sh-TSPAN5 1.10±0.02; Rescue 1.05±0.03).

H) Left panel: Confocal images showing dendrites from DIV18 rat hippocampal cultured neurons transfected at DIV5 with either Scrambled, Sh-TSPAN5 or Rescue constructs all co-expressing GFP immunolabelled for TSPAN5 (in red). A decrease in TSPAN5 signal is seen in Sh-TSPAN5 transfected neurons compared with Scrambled- and Rescue-transfected neurons. Scale bar = 5 µm. Right panel: Quantification of TSPAN5 immunostaining mean intensity (A.U.) (normalized on Scrambled) (Scrambled 1±0.07; Sh-TSPAN5 0.43±0.08; Rescue 0.94±0.12).

I) Sholl analysis of branching points versus distance from the cell body and quantification of the total number of branching point per field of view. Analysis carried out on DIV12 neurons transfected at DIV5 with either Scrambled or Sh-TSPAN5 constructs presented in Fig 2A. No significant differences were found (branching points (N): Scrambled 35±4; Sh-TSPAN5 30±3). N = 20 neurons per condition.

J) Confocal images showing DIV18 rat hippocampal cultured neurons transfected with Scrambled or Sh-TSPAN5 co-expressing GFP and immunolabelled for CamKII α (in red). Similar levels of staining are present in both Scrambled- and Sh-TSPAN5-transfected neurons confirming that these neurons are excitatory (CamKII mean intensity (A.U.): Scrambled 46.6±4.7; Sh-TSPAN5 37.8±2.9). N = 16 neurons per condition. Scale bar = 20 µm.

Values represent the mean ± SEM. * = $p<0.05$, ** = $p<0.01$, *** = $p<0.001$ versus Scrambled; ° = $p<0.05$, °° = $p<0.01$, °°° = $p<0.001$ versus Sh-TSPAN5

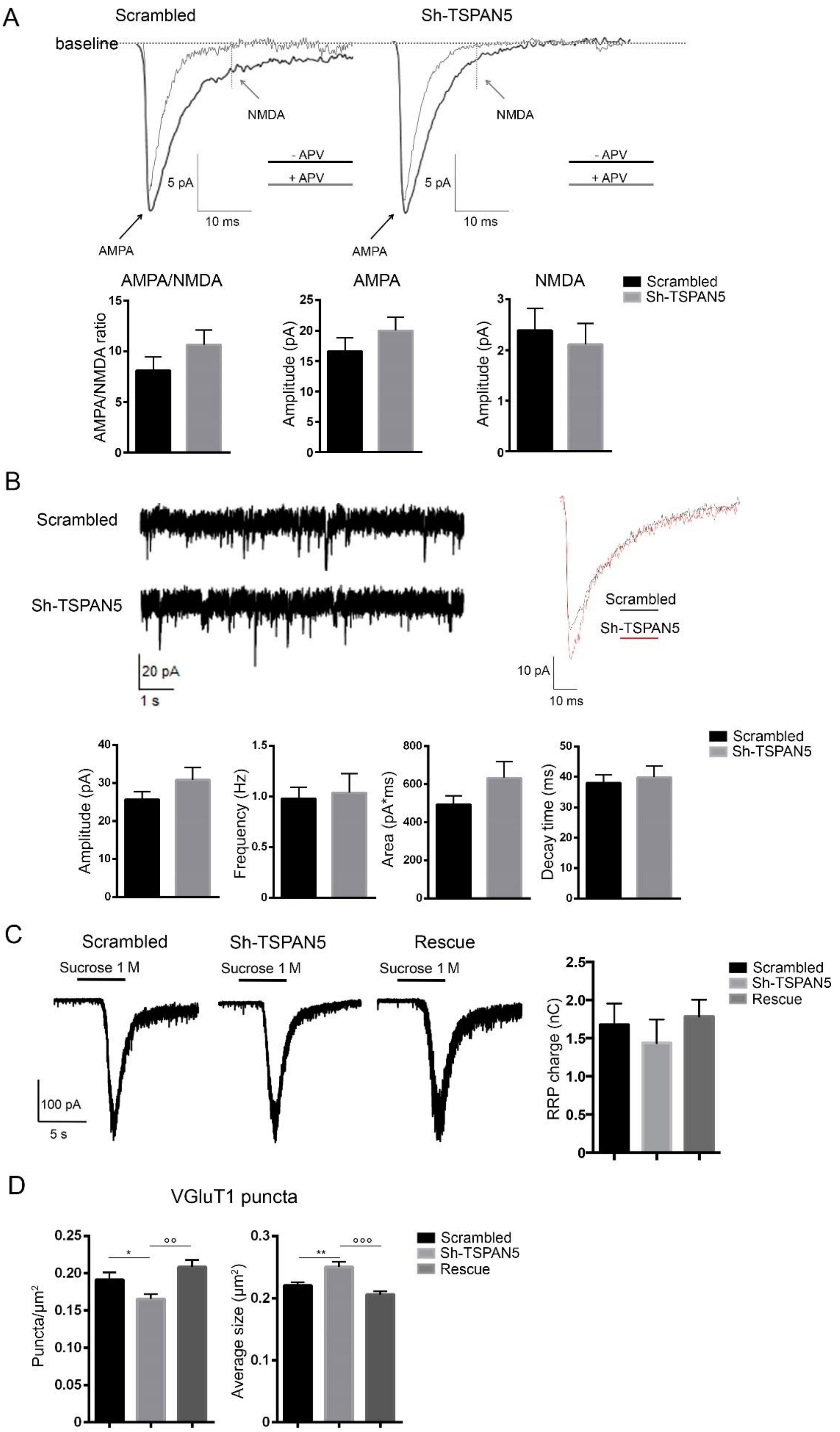

A
Scrambled
Sh-TSPAN5
baseline
NMDA
AMPA
5 pA
10 ms
- APV
+ APV
AMPA/NMDA
AMPA
NMDA
Scrambled
Sh-TSPAN5
AMPA/NMDA ratio
Amplitude (pA)
B
Scrambled
Sh-TSPAN5
20 pA
1 s
10 pA
10 ms
Amplitude (pA)
Frequency (Hz)
Area (pA*ms)
Decay time (ms)
C
Scrambled
Sh-TSPAN5
Rescue
Sucrose 1 M
100 pA
5 s
RRP charge (nC)
D
VGluT1 puncta
Puncta/µm²
Average size (µm²)

Fig S2

Figure S2. Related to figure 3

A) Single representative traces and quantification of the AMPA/NMDA mEPSCs amplitude ratio recorded from DIV18 rat hippocampal cultured neurons transfected at DIV5 with Scrambled or Sh-TSPAN5 constructs. Mixed AMPA/NMDA-mEPSCs were recorded in the absence of Mg^{2+} to reduce Mg^{2+} block of NMDARs. Pure AMPAR currents were isolated by perfusing neurons for at least 6 minutes with the NMDAR blocker APV to estimate the time point of decat of the AMPAR component of mixed AMPA/NMDA-mEPSCs. This allowed the measure of the NMDAR component of mixed AMPA/NMDA-mEPSCs. Specifically, the AMPAR component of mixed events were measured at the peak of the current while the NMDAR component were measured in a window between 10-20 ms after the AMPAR peak. Histograms show no significant differences either in the AMPA/NMDA ratio nor in the two components analysed separately (AMPA/NMDA ratio: Scrambled 8.08±1.37; Sh-TSPAN5 10.62±1.47; AMPA (pA): Scrambled 16.61±2.22; Sh-TSPAN5 20.01±2.17; NMDA (pA): Scrambled 2.39±0.44; Sh-TSPAN5 2.11±0.41). N = Scrambled 7; Sh-TSPAN5 7 neurons.

B) Top panels: Representative traces and single representative traces of mIPSCs recorded from DIV18 rat hippocampal cultured neurons transfected at DIV5 with Scrambled or Sh-TSPAN5 constructs. No significant difference was identified between the analysed conditions.
Bottom panels: quantification of amplitude, frequency, area and decay time of mIPSCs recorded from DIV18 rat hippocampal cultured neurons transfected at DIV5 with Scrambled or Sh-TSPAN5 constructs showing no significant difference in the parameters analysed in Sh-TSPAN5- compared with Scrambled-transfected neurons (amplitude (pA): Scrambled 25.60±2.2; Sh-TSPAN5 30.79±3.24; frequency (Hz): Scrambled 0.98±0.11; Sh-TSPAN5 1.04±0.19; area (pA*ms): Scrambled 493.7±46.2; Sh-TSPAN5 632.7±85.23; decay time (ms): Scrambled 37.98±2.63; Sh-TSPAN5 39.85±3.69). N = Scrambled 14; Sh-TSPAN5 11 neurons.

C) Representative traces and quantification of currents recorded after supplementation of 1 M sucrose in DIV18 rat hippocampal cultured neurons transfected at DIV5 with Scrambled, Sh-TSPAN5 or Rescue constructs showing no difference among the different conditions (RRP charge (nC): Scrambled 1.68±0.28; Sh-TSPAN5 1.50±0.31; Rescue 1.78±0.22). N = Scrambled 10; Sh-TSPAN5 12; Rescue 10 neurons.

D) Histograms showing the quantification of VGluT1 puncta analysed separately, related to Figure 3A. N° of puncta, puncta density and average puncta size showed small differences between the different conditions (puncta/μm^2): Scrambled 0.19±0.01; Sh-TSPAN5 0.17±0.01; Rescue 0.21±0.01; average size (μm^2): Scrambled 0.22±0.01; Sh-TSPAN5 0.25±0.01; Rescue 0.21±0.01). N = 16 neurons/condition

Values represent the mean ± SEM. * = $p<0.05$, ** = $p<0.01$, *** = $p<0.001$ versus Scrambled; ° = $p<0.05$, °° = $p<0.01$, °°° = $p<0.001$ versus Sh-TSPAN5

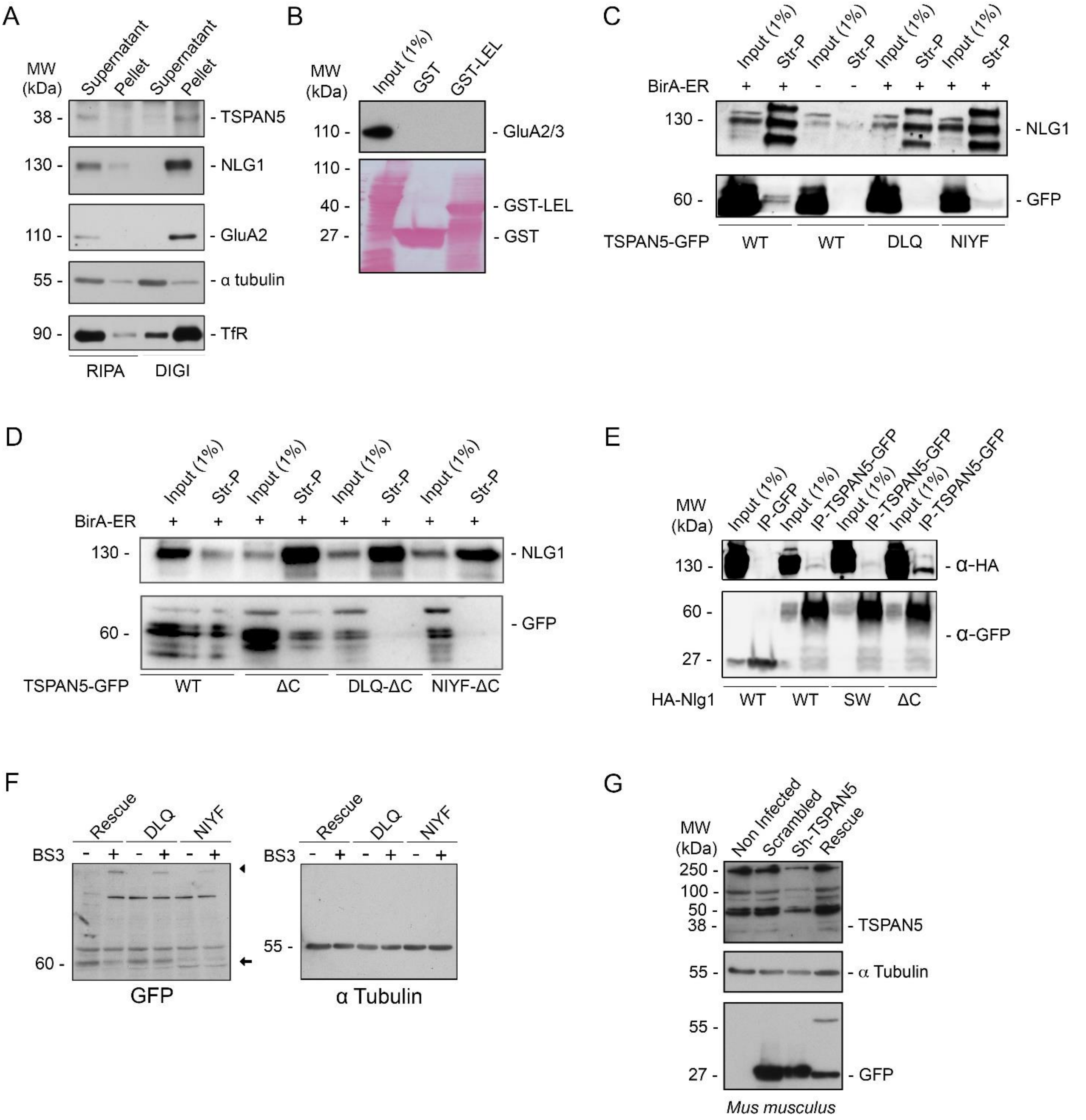
A
MW (kDa)
Supernatant
Pellet
Supernatant
Pellet
38 -
130 -
110 -
55 -
90 -
- TSPAN5
- NLG1
- GluA2
- α tubulin
- TfR
RIPA
DIGI
B
MW (kDa)
Input (1%)
GST
GST-LEL
110 -
- GluA2/3
110 -
40 -
27 -
- GST-LEL
- GST
C
Input (1%)
Str-P
BirA-ER
130 -
60 -
- NLG1
- GFP
TSPAN5-GFP
WT
WT
DLQ
NIYF
D
Input (1%)
Str-P
BirA-ER
130 -
60 -
- NLG1
- GFP
TSPAN5-GFP
WT
ΔC
DLQ-ΔC
NIYF-ΔC
E
MW (kDa)
Input (1%)
IP-GFP
IP-TSPAN5-GFP
130 -
60 -
27 -
- α-HA
- α-GFP
HA-Nlg1
WT
WT
SW
ΔC
F
Rescue
DLQ
NIYF
BS3
60 -
GFP
55 -
α Tubulin
G
MW (kDa)
Non Infected
Scrambled
Sh-TSPAN5
Rescue
250 -
100 -
50 -
38 -
55 -
55 -
27 -
- TSPAN5
- α Tubulin
- GFP
Mus musculus

Fig S3

Figure S3. Related to Figure 4-5

A) Western blot representing rat hippocampal cultured neurons lysed at DIV12 with RIPA buffer (containing Triton X-100 and NP40) or with buffer containing 1% digitonin. Supernatant or pellet fractions are shown. TSPAN5, neuroligin-1 and GluA2 were enriched in the supernatant in RIPA lysates and in the pellets in the digitonin containing buffer. Transferrin Receptor (TfR) was used as a plasma membrane control protein and displayed a more diffuse distribution. Tubulin, used as a cytosolic control protein, was enriched in the supernatant fraction independently of the detergent used.
B) Western blot showing GST-pulldown experiment on adult rat hippocampus and cortex lysates in RIPA using empty GST or GST fused to LEL (GST-LEL). The input was 2.5% of pulldown volume. No GluA2 binding to GST-LEL was observed.
C) Western blot showing Streptavidin precipitation experiment on transfected HEK293 cells lysed with RIPA buffer. All cells were transfected with AP-neuroligin-1, with or without BirA-ER (negative control) and with either TSPAN5-GFP wild-type or TSPAN5-GFP mutated at the DLQ or NIYF residues. Streptavidin coupled to agarose beads (Str-P) was used to specifically precipitate biotinylated AP-neuroligin-1. α-NLG1 or α-GFP antibodies were used to visualize precipitated protein. Note that both neuroligin-1 and TSPAN5-GFP are absent in the Str-P lane when BirA-ER was not transfected. Precipitation of AP-neuroligin-1 co-precipitated TSPAN5-GFP wild-type whereas the signal is almost undetectable for both TSPAN5-DLQ-GFP and TSPAN5-NIYF-GFP. Input was 1% of the precipitated volume.
D) Western blot showing Streptavidin precipitation experiment on transfected HEK293 cells lysed with RIPA buffer. All cells were transfected with AP-neuroligin-1, BirA-ER and with either wild-type TSPAN5-GFP, TSPAN5-ΔC-GFP mutant or the double mutants DLQ-ΔC or NIYF-ΔC. Streptavidin coupled to agarose beads (Str-P) was used specifically to precipitate biotinylated AP-neuroligin-1. α-NLG1 or α-GFP antibodies were used to visualize precipitated protein. Precipitation of AP-neuroligin-1 co-precipitated TSPAN5-GFP wild-type and to a lesser extent TSPAN5-ΔC-GFP whereas the signal is almost undetectable for both TSPAN5-DLQ-ΔC-GFP and TSPAN5-NIYF-ΔC-GFP. Input was 1% of the precipitated volume.
E) Western blot showing GFP-trap precipitation experiments using RIPA lysates of HEK293 cells co-transfected with either cytosolic GFP or TSPAN5-GFP, plus HA-tagged wild type neuroligin-1 (WT), SWAP mutant (SW) or delta-C mutant (ΔC) neuroligin-1. α-HA or α-GFP antibodies were used to visualize precipitated proteins. The HA signal is evident in the IP-TSPAN5-GFP lanes with co-transfection of both WT and ΔC mutant neuroligin-1. In the SWAP mutant co-transfected cells, the HA signal is reduced. Input was 1% of the precipitated volume.
F) Western blot showing BS3 crosslinking experiment on DIV14 rat hippocampal cultured neurons infected at DIV5 with lentiviral particles carrying either Rescue, DLQ or NIYF

TSPAN5 cDNA all fused to GFP. The total and intracellular TSPAN5 are visible at 60 kDa (arrow), whereas the crosslinked TSPAN5 is visible at the top of the blot only in the BS3 + lanes (arrowhead). Tubulin is used as loading control and to verify the integrity of the cultures.

G) Western blot showing TSPAN5 immunoreactive band in lysates in BS3 buffer from DIV14 mouse hippocampal cultured neurons non-infected or infected at DIV5 with lentiviral particles carrying Scrambled, Sh-TSPAN5 or Rescue DNA. A reduction of intensity of the band detected by α-TSPAN5 antibody was observed in the Sh-TSPAN5 lane. Tubulin is used as loading control and GFP as control for infection. Note that the Rescue construct produces both GFP fused TSPAN5 (around 60 kDa) and soluble GFP.

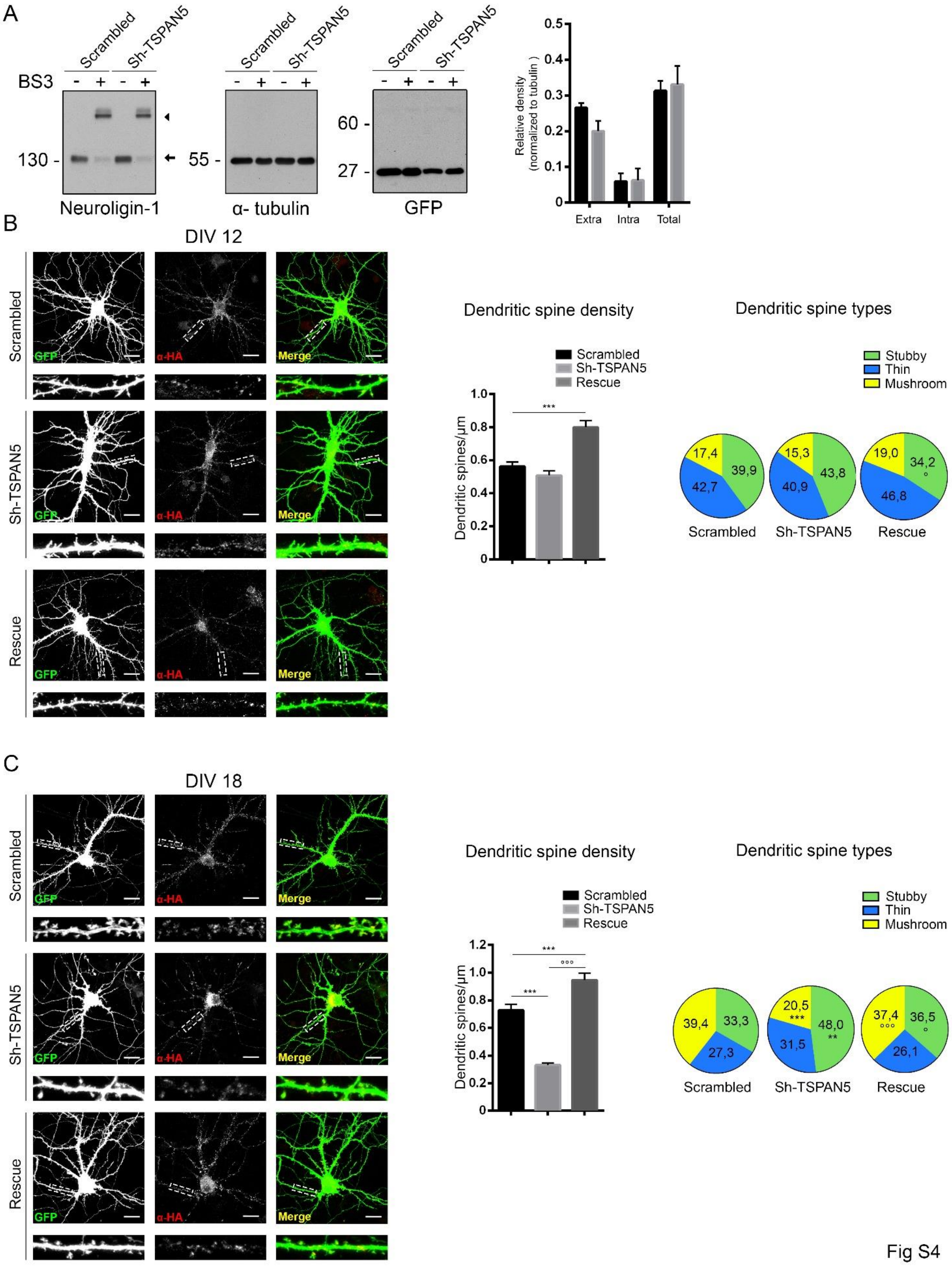

A
Scrambled
Sh-TSPAN5
BS3
- + - +
130 -
55 -
60 -
27 -
Neuroligin-1
α- tubulin
GFP
Relative density
(normalized to tubulin)
0.5
0.4
0.3
0.2
0.1
0
Extra
Intra
Total
B
DIV 12
Scrambled
Sh-TSPAN5
Rescue
GFP
α-HA
Merge
Dendritic spine density
Scrambled
Sh-TSPAN5
Rescue
Dendritic spines/µm
1.0
0.8
0.6
0.4
0.2
0
***
Dendritic spine types
Stubby
Thin
Mushroom
17,4
39,9
42,7
15,3
43,8
40,9
19,0
34,2
°
46,8
Scrambled
Sh-TSPAN5
Rescue
C
DIV 18
Scrambled
Sh-TSPAN5
Rescue
GFP
α-HA
Merge
Dendritic spine density
Scrambled
Sh-TSPAN5
Rescue
Dendritic spines/µm
1.2
1.0
0.8
0.6
0.4
0.2
0
***
°°°
***
Dendritic spine types
Stubby
Thin
Mushroom
39,4
33,3
27,3
20,5
***
48,0
**
31,5
37,4
°°°
36,5
°
26,1
Scrambled
Sh-TSPAN5
Rescue

Fig S4

Fig S4. Related to Figure 6

A) Western blot of the BS3 crosslinking experiment in DIV12 rat hippocampal cultured neurons infected at DIV5 with lentiviral particles carrying Scrambled or Sh-TSPAN5 DNA to evaluate neuroligin-1 levels. Total and intracellular neuroligin-1 was detected at 130 kDa (indicated by arrow), whereas surface neuroligin-1 was visible at a higher molecular weight only in BS3 + lanes (indicated by arrowhead). No change was observed. GFP was used as control for infection and Tubulin as both a loading control and to verify the integrity of BS3 crosslinking.

B) Left panels: Confocal images and quantification of DIV12 rat hippocampal cultured neurons transfected at DIV5 with HA-neuroligin-1 and either Scrambled, Sh-TSPAN5 or Rescue constructs all co-expressing GFP and immunolabelled with an α-HA antibody. Inserts show higher magnification of the dendrites highlighted in white. Scale bar = 20 µm.

   Middle panels: Quantification of the dendritic spine density (dendritic spine/µm: Scrambled 0.56±0.03; Sh-TSPAN5 0.51±0.03; Rescue 0.80±0.04). Differences were only observed in the Rescue condition.

   Right panel: Quantification of dendritic spine morphology analysis represented as percentages of spines divided in three categories: Stubby, Thin and Mushroom (pie charts) (Stubby (%): Scrambled 39.89±3.24; Sh-TSPAN5 43.85±2.85; Rescue 34.21±1.60. Thin (%): Scrambled 42.72±3.55; Sh-TSPAN5 40.88±2.49; Rescue 46.75±1.99. Mushroom (%): Scrambled 17.39±1.38; Sh-TSPAN5 15.28±1.43; Rescue 19.04±1.32). N = Scrambled, 16; Sh-TSPAN5, 19; Rescue, 15 neurons.

C) Left panels: Confocal images and quantification of DIV18 rat hippocampal cultured neurons transfected at DIV5 with HA-neuroligin-1 and either Scrambled, Sh-TSPAN5 or Rescue constructs all co-expressing GFP and immunolabelled with an α-HA antibody. Inserts show higher magnification of the dendrites highlighted in white. Scale bar = 20 µm.

   Middle panels: Quantification of the dendritic spine density (dendritic spine/µm: Scrambled 0.73±0.04; Sh-TSPAN5 0.33±0.02; Rescue 0.95±0.05). There is a significant reduction of dendritic spine density in Sh-TSPAN5-transfected neurons compared with Scrambled and Rescue-transfected neurons.

   Right panel: Quantification of the dendritic spine morphology analysis showed as percentages of spines divided in three categories: Stubby, Thin and Mushroom (pie charts). Sh-TSPAN5-transfected neurons showed a significant increase in the percentage of Stubby spines, at the expense of Mushroom spines, compared with Scrambled. Rescue-transfected neurons displayed an increase in Mushroom spines and a decrease in Stubby spines compared with Sh-TSPAN5 (Stubby (%): Scrambled

33.3±2.7; Sh-TSPAN5 48.0±4.6; Rescue 36.5±1.8. Thin (%): Scrambled 27.3±1.8; Sh-TSPAN5 31.5±4.3; Rescue 26.1±1.5. Mushroom (%): Scrambled 39.4±2.9; Sh-TSPAN5 20.5±2.6; Rescue 37.4±1.7). N = Scrambled, 15; Sh-TSPAN5, 14; Rescue, 13 neurons.

Values represent the mean ± SEM. * = p<0.05, ** = p<0.01, *** = p<0.001 versus Scrambled; ° = p<0.05, °° = p<0.01, °°° = p<0.001 versus Sh-TSPAN5

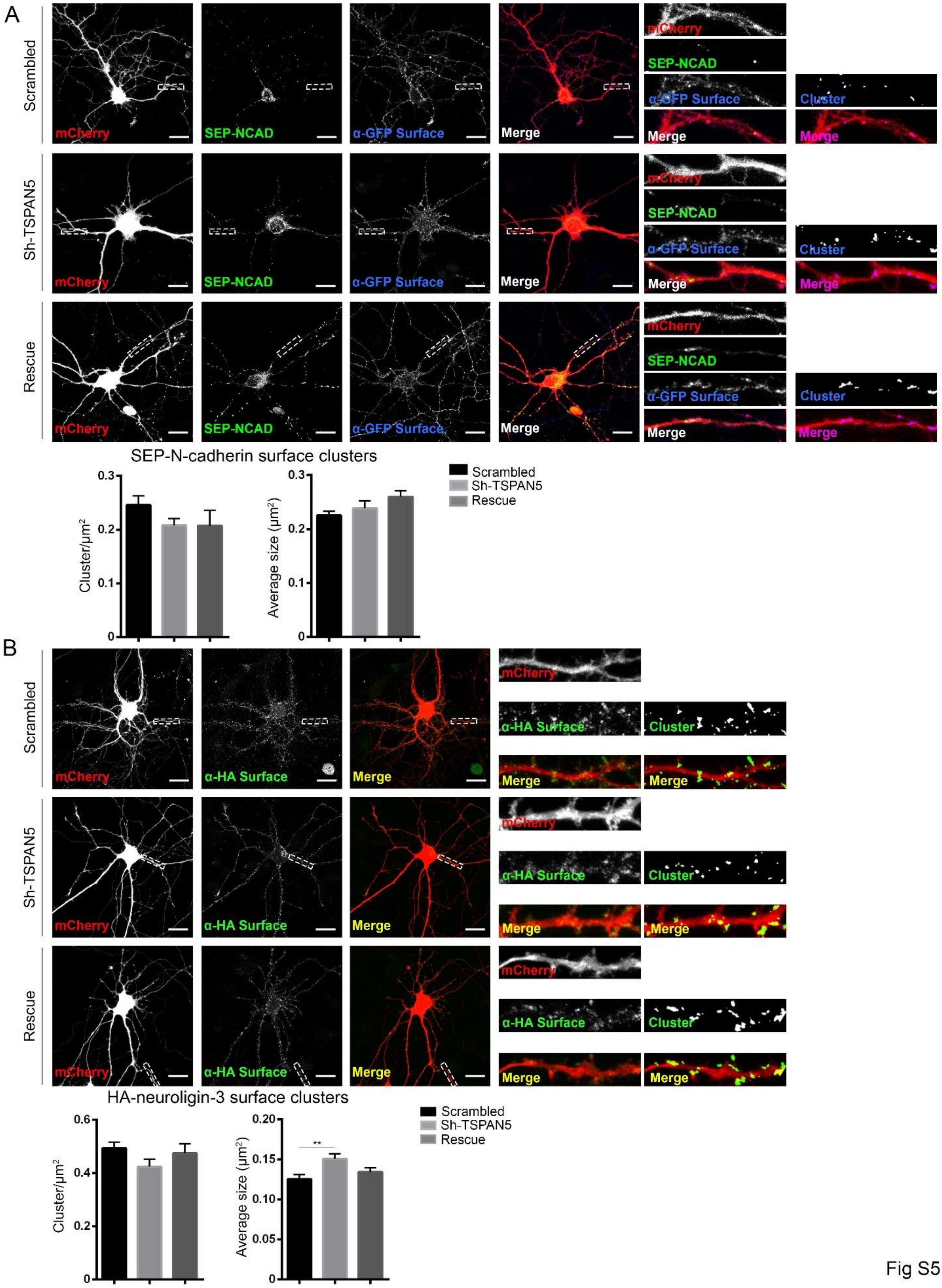
A
Scrambled
Sh-TSPAN5
Rescue
mCherry
SEP-NCAD
α-GFP Surface
Merge
Cluster
SEP-N-cadherin surface clusters
Cluster/μm²
Average size (μm²)
Scrambled
Sh-TSPAN5
Rescue
B
α-HA Surface
HA-neuroligin-3 surface clusters
**

Fig S5

Figure S5. Related to Figure 6

A) Top panels: Confocal images of dendrites from DIV12 rat hippocampal cultured neurons transfected at DIV5 with SEP-N-cadherin and either Scrambled, Sh-TSPAN5 or Rescue constructs all co-expressing mCherry. The signal generated from surface-applied α-GFP antibody and the subsequent cluster analysis are shown in blue. Inserts show higher magnification of the dendrites highlighted in white. Scale bar = 20µm.

   Bottom panels: Quantification of the cluster density and average size of the clusters. No significant difference was detected (SEP-N-Cadherin: cluster/μm^2): Scrambled 0.25±0.02; Sh-TSPAN5 0.21±0.01; Rescue 0.21±0.03; average size (μm^2): Scrambled 0.23±0.01; Sh-TSPAN5 0.24±0.01; Rescue 0.26±0.01). N = Scrambled 19, Sh-TSPAN5 22, Rescue 22 neurons.

B) Top panels: Confocal images of dendrites from DIV12 rat hippocampal cultured neurons transfected at DIV5 with HA-neuroligin-3 and either Scrambled, Sh-TSPAN5 or Rescue constructs all co-expressing mCherry. The signal generated from surface-applied α-HA antibody and subsequent clusters analysis are shown in green. Inserts show higher magnification of the dendrites highlighted in white. Scale bar = 20µm.

   Bottom panels: Quantification of the cluster density and average size of the clusters. A small significant increase in the average size of clusters in Sh-TSPAN5-transfected neurons was detected (HA-neuroligin-3: cluster/μm^2: Scrambled 0.49±0.02; Sh-TSPAN5 0.42±0.03; Rescue 0.48±0.04; average size (μm^2): Scrambled 0.13±0.01; Sh-TSPAN5 0.15±0.01; Rescue 0.13±0.01). N = Scrambled 23, Sh-TSPAN5 22, Rescue 19 neurons.

Values represent the mean ± SEM. * = p<0.05, ** = p<0.01, *** = p<0.001 versus Scrambled; ° = p<0.05, °° = p<0.01, °°° = p<0.001 versus Sh-TSPAN5

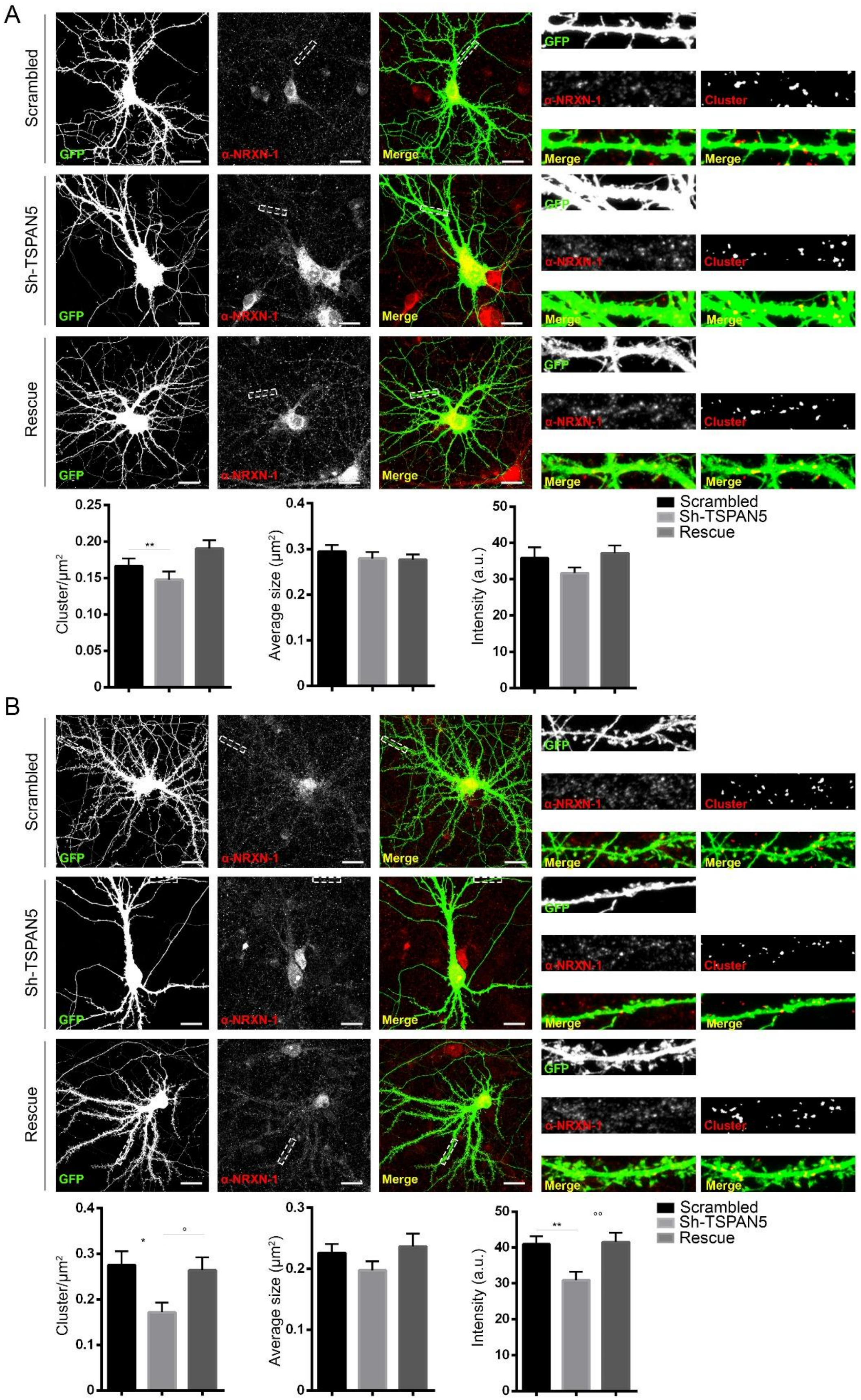
A
Scrambled
Sh-TSPAN5
Rescue
GFP
α-NRXN-1
Merge
Cluster
Cluster/µm²
Average size (µm²)
Intensity (a.u.)
**
Scrambled
Sh-TSPAN5
Rescue
B
*
°
°°
0.25
0.20
0.15
0.10
0.05
0
0.4
0.3
0.2
0.1
50
40
30
20
10

Fig S6

Figure S6. Related to Figure 6

A) Top panels: Confocal images of dendrites from DIV12 rat hippocampal cultured neurons transfected at DIV5 with Scrambled, Sh-TSPAN5 or Rescue constructs all co-expressing GFP. α-pan neurexin-1 immunolabelling and clusters are shown in red. Inserts show higher magnification of the dendrites highlighted in white. Scale bar = 20µm.

   Bottom panels: Quantification of the cluster density, average size of the clusters and intensity of the α-pan neurexin-1 immunolabelling signal. A small significant decrease of cluster density in Sh-TSPAN5-transfected neurons was detected (cluster/μm^2: Scrambled 0.17±0.01; Sh-TSPAN5 0.15±0.01; Rescue 0.19±0.03; average size (μm^2): Scrambled 0.29±0.01; Sh-TSPAN5 0.28±0.01; Rescue 0.28±0.01; intensity (arbitrary units): Scrambled 35.83±3.01; Sh-TSPAN5 31.69±1.53; Rescue 37.19±2.13). N = Scrambled 19, Sh-TSPAN5 19, Rescue 15 neurons.

B) Top panels: Confocal images of dendrites from DIV18 rat hippocampal cultured neurons transfected at DIV5 with Scrambled, Sh-TSPAN5 or Rescue constructs all co-expressing GFP. α-pan neurexin-1 immunolabelling and clusters are shown in red. Inserts show higher magnification of the dendrites highlighted in white. Scale bar = 20µm.

   Bottom panels: Quantification of the cluster density, average size of the clusters and intensity of the α-pan neurexin-1 immunolabelling signal. A significant decrease was observed for both cluster density and signal intensity in Sh-TSPAN5-transfected neurons compared with Scrambled-transfected neurons (cluster/μm^2: Scrambled 0.27±0.03; Sh-TSPAN5 0.17±0.02; Rescue 0.26±0.03; average size (μm^2): Scrambled 0.23±0.01; Sh-TSPAN5 0.20±0.01; Rescue 0.24±0.02; intensity (arbitrary units): Scrambled 40.89±2.26; Sh-TSPAN5 30.88±2.33; Rescue 41.52±2.62). N = Scrambled 20, Sh-TSPAN5 20, Rescue 19 neurons.

Values represent the mean ± SEM. * = p<0.05, ** = p<0.01, *** = p<0.001 versus Scrambled; ° = p<0.05, °° = p<0.01, °°° = p<0.001 versus Sh-TSPAN5

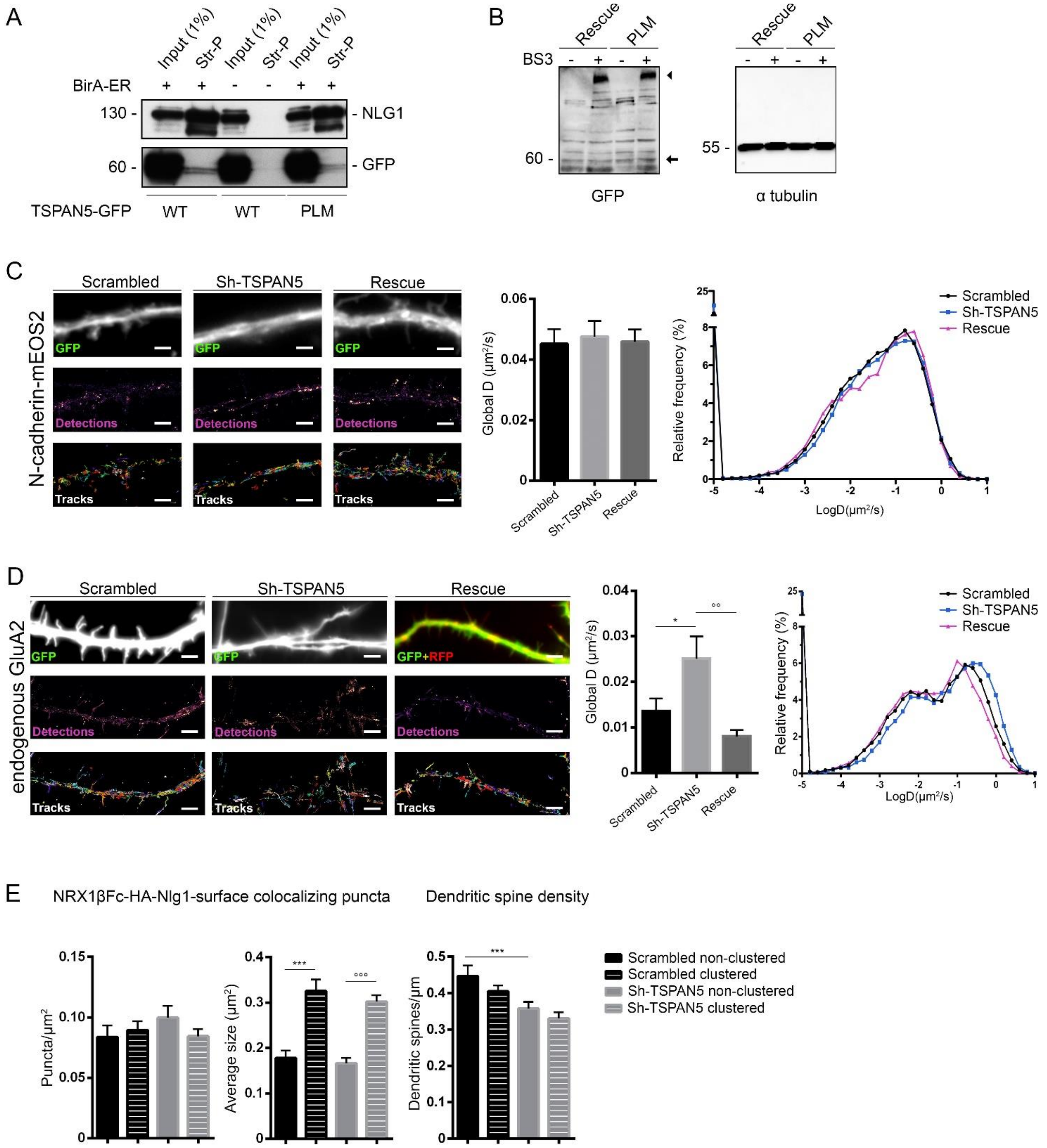
A
Input (1%)
Str-P
BirA-ER
130 -
60 -
- NLG1
- GFP
TSPAN5-GFP
WT
PLM
B
Rescue
PLM
BS3
60 -
55 -
GFP
α tubulin
C
Scrambled
Sh-TSPAN5
Rescue
N-cadherin-mEOS2
GFP
Detections
Tracks
Global D (μm²/s)
Relative frequency (%)
LogD(μm²/s)
D
endogenous GluA2
GFP+RFP
E
NRX1βFc-HA-Nlg1-surface colocalizing puncta
Dendritic spine density
Puncta/μm²
Average size (μm²)
Dendritic spines/μm
Scrambled non-clustered
Scrambled clustered
Sh-TSPAN5 non-clustered
Sh-TSPAN5 clustered

Fig S7

Figure S7. Related to Figure 6

A) Western blot showing Streptavidin precipitation experiment on transfected HEK293 cells lysed in RIPA buffer. Cells were transfected with AP-neuroligin-1, with or without BirA-ER (negative control) and with either TSPAN5-GFP wild-type or TSPAN5-PLM-GFP mutant. Streptavidin coupled to agarose beads (Str-P) was used specifically to precipitate biotinylated AP-neuroligin-1. α-NLG1 or α-GFP antibodies were used to visualize precipitated protein. Note that both neuroligin-1 and TSPAN5-GFP are absent in the Str-P lane when BirA-ER was not transfected. Precipitation of AP-neuroligin-1 co-precipitated both TSPAN5-GFP wild-type and TSPAN5-PLM-GFP. Input was 1% of the precipitated volume.

B) Western blot showing BS3 crosslinking experiment on DIV14 rat hippocampal cultured neurons infected at DIV5 with lentiviral particles carrying either Rescue or PLM TSPAN5 cDNA all fused to GFP. The total and intracellular TSPAN5 are visible at 60 kDa (arrow), whereas the crosslinked TSPAN5 is visible at the top of the blot only in the BS3 + lane (arrowhead). Tubulin is used as loading control and to verify the integrity of the cultures.

C) Left panels: Single molecule tracking experiments on mEOS-N-cadherin. Images represent DIV12 rat hippocampal cultured neurons transfected at DIV5 with both mEOS-N-Cadherin and either Scrambled, Sh-TSPAN5 or Rescue constructs all co-expressing GFP. Top imaging panel: Widefield GFP signal of imaged dendrites. Middle imaging panel: Super-resolved activated mEOS detection maps generated from 4,000 frames with a 20-ms integration time shown as an intensity scale. Bottom imaging panel: mEOS-N-cadherin trajectories calculated from stacks of 4,000 images with a 20-ms exposure time shown in pseudocolors. Scale bar = 2 µm.

   Middle panel: Quantification of single molecule tracking demonstrating no changes of mEOS-N-Cadherin mobility (global diffusion coefficient ($\mu m^2/s$): Scrambled 0.045±0.005; Sh-TSPAN5 0.048±0.005; Rescue 0.046±0.004).

   Right panel: Logarithmic distribution plot of diffusion coefficients for Scrambled- (black) Sh-TSPAN5- (blue) or Rescue-transfected (purple) neurons.

   N = Scrambled 18, Sh-TSPAN5 19, Rescue 16 neurons.

D) Left panels: Single molecule tracking experiments of GluA2. Images represent DIV12 rat hippocampal cultured neurons transfected at DIV5 with either Scrambled or Sh-TSPAN5 both co-expressing GFP or with Rescue co-expressing RFP plus Sh-TSPAN5 co-expressing GFP. Top imaging panel: Widefield GFP or GFP + RFP signal of imaged dendrites. Middle imaging panel: Super-resolved GluA2 detection maps generated from 4,000 frames with a 20-ms integration time shown as an intensity scale. Bottom imaging panel: GluA2 trajectories calculated from stacks of 4,000 images with a 20-ms exposure time shown in pseudocolors. Scale bar = 2 µm.

Middle panel: Quantification of single molecule tracking demonstrating an increased mobility of GluA2 in Sh-TSPAN5-transfected neurons and the restoration of the phenotype in Rescue-transfected neurons (global diffusion coefficient ($\mu m^2/s$): Scrambled 0.013±0.002; Sh-TSPAN5 0.025±0.005; Rescue 0.008±0.001).

Right panel: Logarithmic distribution plot of diffusion coefficients for Scrambled- (black) Sh-TSPAN5- (blue) or Rescue-transfected (purple) neurons.

N = Scrambled 22, Sh-TSPAN5 21, Rescue 18 neurons.

E) Left histograms: Quantification of the neuroligin-1/neurexin1β-Fc colocalizing cluster density and average cluster size demonstrating that the treatment with pre-clustered neurexin1β-Fc was sufficient to increase the size of clusters of neuroligin-1 without affecting its density (puncta/μm^2: Scrambled non-clustered 0.08±0.01; Scrambled clustered 0.09±0.01; Sh-TSPAN5 non-clustered 0.10±0.01; Sh-TSPAN5 clustered 0.08±0.01; average size (μm^2): Scrambled non-clustered 0.18±0.02; Scrambled clustered 0.33±0.03; Sh-TSPAN5 non-clustered 0.17±0.01; Sh-TSPAN5 clustered 0.30±0.01).

Right histogram: Quantification of dendritic spine density showed that the treatment with pre-clustered neurexin1β-Fc had no effect on spine density. Sh-TSPAN5 transfected neurons treated with the non-clustered neurexin1β-Fc shows a reduction compared to Scrambled neurons (dendritic spines/µm: Scrambled non-clustered 0.45±0.03; Scrambled clustered 0.41±0.02; Sh-TSPAN5 non-clustered 0.36±0.02; Sh-TSPAN5 clustered 0.33±0.02).

N = Scrambled non-clustered 10, Scrambled clustered 14, Sh-TSPAN5 non-clustered 16, Sh-TSPAN5 clustered 14.

Values represent the mean ± SEM. * = p<0.05, ** = p<0.01, *** = p<0.001 versus Scrambled; ° = p<0.05, °° = p<0.01, °°° = p<0.001 versus Sh-TSPAN5.

Table S1. Exact values and N related to Figures 1-6.

Figure	Panel	Dimension	Values (Mean±SEM)	N
**1**	**C**	percentage of TSPAN5 puncta colocalizing with (%)	PSD-95: 87±1; GluA2: 85±3; Bassoon: 28±3; VGluT1: 8±2; VGAT: 5±1; $GABA_AR$-β3: 13±2	10 neurons
**2**	**B**	dendritic spine/μm	Scrambled 0.27±0.01; Sh-TSPAN5 0.24±0.01; Rescue 0.29±0.02	Scrambled, 25; Sh-TSPAN5, 26; Rescue, 22 neurons.
**2**	**B**	Stubby (%)	Scrambled 33.24±2.00; Sh-TSPAN5 38.59±2.00; Rescue 41.68±2.00	Scrambled, 25; Sh-TSPAN5, 26; Rescue, 22 neurons.
**2**	**B**	Thin (%)	Scrambled 48.85±2.00; Sh-TSPAN5 46.60±2.00; Rescue 37.69±2.00	Scrambled, 25; Sh-TSPAN5, 26; Rescue, 22 neurons.
**2**	**B**	Mushroom (%)	Scrambled 17.87±1; Sh-TSPAN5 14.80±1.00; Rescue 20.63±1.00	Scrambled, 25; Sh-TSPAN5, 26; Rescue, 22 neurons.
**2**	**D**	dendritic spine/μm	Scrambled 0.24±0.01; Sh-TSPAN5 0.17±0.01; Rescue 0.26±0.02	Scrambled, 19; Sh-TSPAN5, 20; Rescue, 20 neurons
**2**	**D**	Stubby (%)	Scrambled 40.4±1.5; Sh-TSPAN5 51.7±1.9; Rescue 37.5±1.7	Scrambled, 19; Sh-TSPAN5, 20; Rescue, 20 neurons
**2**	**D**	Thin (%)	Scrambled 18.8±1.4; Sh-TSPAN5 23.1±1.4; Rescue 18.2±1.0	Scrambled, 19; Sh-TSPAN5, 20; Rescue, 20 neurons
**2**	**D**	Mushroom (%)	Scrambled 40.8±1.3; Sh-TSPAN5 25.1±1.7; Rescue 44.2±2.1	Scrambled, 19; Sh-TSPAN5, 20; Rescue, 20 neurons
**2**	**F**	dendritic spine/μm	Scrambled 0.55±0.02; Sh-TSPAN5 0.22±0.02; Rescue 0.57±0.03	Scrambled, 16; Sh-TSPAN5, 16; Rescue, 16 neurons
**2**	**F**	Stubby (%)	Scrambled 41.0±1.8; Sh-TSPAN5 59.2±2.9; Rescue 35.5±2.1	Scrambled, 16; Sh-TSPAN5, 16; Rescue, 16 neurons
**2**	**F**	Thin (%)	Scrambled 20.7±1.4; Sh-TSPAN5 19.3±2.7; Rescue 20.3±2.0	Scrambled, 16; Sh-TSPAN5, 16; Rescue, 16 neurons
**2**	**F**	Mushroom (%)	Scrambled 38.3±1.6; Sh-TSPAN5 21.5±2.0; Rescue 44.3±1.5	Scrambled, 16; Sh-TSPAN5, 16; Rescue, 16 neurons
**2**	**G**	dendritic spine/μm	Scrambled DIV12 0.048±0.004; Scrambled DIV14 0.099±0.005; Scrambled DIV18 0.207±0.009; Sh-TSPAN5 DIV12 0.033±0.003; Sh-TSPAN5 DIV14 0.045±0.005; Sh-TSPAN5 DIV18 0.048±0.006; Rescue DIV12 0.065±0.008; Rescue DIV14 0.133±0.012; Rescue DIV18 0.253±0.014	As in Fig 2B, D, F
**2**	**H**	dendritic spine/μm	Scrambled DIV12 0.22±0.01; Scrambled DIV14 0.15±0.01; Scrambled DIV18 0.34±0.02; Sh-TSPAN5 DIV12 0.19±0.01; Sh-TSPAN5 DIV14 0.13±0.01; Sh-	As in Fig 2B, D, F

			TSPAN5 DIV18 0.18±0.02; Rescue DIV12 0.23±0.02; Rescue DIV14 0.15±0.01; Rescue 0.32±0.02	
**3**	**B**	N° of puncta	Scrambled 259±17; Sh-TSPAN5 188±15; Rescue 242±18	16 neurons/condition
**3**	**B**	puncta/µm2	Scrambled 0.117±0.005; Sh-TSPAN5 0.105±0.005; Rescue 0.108±0.005	16 neurons/condition
**3**	**B**	average size (µm^2)	Scrambled 0.144±0.005; Sh-TSPAN5 0.155±0.006; Rescue 0.143±0.006	16 neurons/condition
**3**	**B**	puncta on dendritic spines/total (%)	Scrambled 55.7±2.4; Sh-TSPAN5 29.8±3.3; Rescue 53.2±2.0	16 neurons/condition
**3**	**B**	puncta on dendritic shaft/total (%)	Scrambled 31.88±2.95; Sh-TSPAN5 46.54±4.38; Rescue 30.98±2.75	16 neurons/condition
**3**	**B**	puncta on soma/total (%)	Scrambled 12.87±1.73; Sh-TSPAN5 24.60±2.59; Rescue 16.37±2.41	16 neurons/condition
**3**	**B**	Stubby (%)	Scrambled 27.8±1.3; Sh-TSPAN5 53.3±4.9; Rescue 26.1±1.4	16 neurons/condition
**3**	**B**	Thin (%)	Scrambled 6.2±0.8; Sh-TSPAN5 5.4±1.6; Rescue 5.8±1	16 neurons/condition
**3**	**B**	Mushroom (%)	Scrambled 66.1±1.3; Sh-TSPAN5 41.3±4.7; Rescue 68.1±1.2	16 neurons/condition
**3**	**D**	amplitude (pA)	Scrambled 14.03±0.68; Sh-TSPAN5 13.92±0.70; Rescue 15.37±0.61	Scrambled 25; Sh-TSPAN5 26; Rescue 19 neurons
**3**	**D**	frequency (Hz)	Scrambled 1.17±0.11; Sh-TSPAN5 0.95±0.12; Rescue 1.28±0.15	Scrambled 25; Sh-TSPAN5 26; Rescue 19 neurons
**3**	**D**	area (pA*ms)	Scrambled 97.58±7.07; Sh-TSPAN5 68.47±5.48; Rescue 103.80±7.82	Scrambled 25; Sh-TSPAN5 26; Rescue 19 neurons
**3**	**D**	decay time (ms)	Scrambled 11.96±0.73; Sh-TSPAN5 8.46±0.59; Rescue 11.50±0.76	Scrambled 25; Sh-TSPAN5 26; Rescue 19 neurons
**4**	**F**	Colocalization (%)	82.35±7.75	4 neurons
**4**	**F**	Area of overlap (%)	60.38±6.32	4 neurons
**5**	**A**	dendritic spine/µm	Scrambled 0.64±0.03; Sh-TSPAN5 0.45±0.03: Rescue 0.52±0.03	Scrambled, 17; Sh-TSPAN5, 19; Rescue, 15 neurons
**5**	**A**	Stubby (%)	Scrambled 29.1±1.97; Sh-TSPAN5 47.97±2.86; Rescue 40.56±2.55	Scrambled, 17; Sh-TSPAN5, 19; Rescue, 15 neurons
**5**	**A**	Thin (%)	Scrambled 44.73±1.83; Sh-TSPAN5 33.62±2.65; Rescue 32.06±3.24	Scrambled, 17; Sh-TSPAN5, 19; Rescue, 15 neurons
**5**	**A**	Mushroom (%)	Scrambled 26.17±2.44; Sh-TSPAN5 20.62±2.03; Rescue 27.38±2.03	Scrambled, 17; Sh-TSPAN5, 19; Rescue, 15 neurons
**5**	**B**	dendritic spine/µm	Scrambled 0.72±0.03; Sh-TSPAN5 0.6±0.02: Rescue 0.66±0.03	Scrambled, 19; Sh-TSPAN5, 17; Rescue, 17 neurons

**5**	**B**	Stubby (%)	Scrambled 29.46±1.11; Sh-TSPAN5 45.84±3.08; Rescue 49.44±2.28	Scrambled, 19; Sh-TSPAN5, 17; Rescue, 17 neurons
**5**	**B**	Thin (%)	Scrambled 31.41±2.52; Sh-TSPAN5 40.18±0.03; Rescue 34.08±2.21	Scrambled, 19; Sh-TSPAN5, 17; Rescue, 17 neurons
**5**	**B**	Mushroom (%)	Scrambled 16.35±1.19; Sh-TSPAN5 13.97±0.01; Rescue 16.49±2.21	Scrambled, 19; Sh-TSPAN5, 17; Rescue, 17 neurons
**5**	**C**	dendritic spine/µm	Scrambled 0.77±0.04; Sh-TSPAN5 0.34±0.02; Rescue 0.70± 0.02; DLQ 0.45± 0.03; NIYF 0.20± 0.04	Scrambled, 16; Sh-TSPAN5, 14; Rescue, 16; DLQ, 16; NIYF, 16 neurons
**5**	**C**	Stubby (%)	Scrambled 28.4±2.0; Sh-TSPAN5 48.6±3.1; Rescue 31.1±1.9; DLQ 38.7±4.4; NIYF 54.4±6.0	Scrambled, 16; Sh-TSPAN5, 14; Rescue, 16; DLQ, 16; NIYF, 16 neurons
**5**	**C**	Thin (%)	Scrambled 26.7±2.0; Sh-TSPAN5 32.9±2.9; Rescue 23.3±1.4; DLQ 30.7±2.8; NIYF 33.0±5.2	Scrambled, 16; Sh-TSPAN5, 14; Rescue, 16; DLQ, 16; NIYF, 16 neurons
**5**	**C**	Mushroom (%)	Scrambled 44.8±1.4; Sh-TSPAN5 18.5±1.7; Rescue 45.6±1.8; DLQ 30.6±3.1; NIYF 12.7±3.0	Scrambled, 16; Sh-TSPAN5, 14; Rescue, 16; DLQ, 16; NIYF, 16 neurons
**5**	**D**	dendritic spine/µm	Scrambled 1.51±0.04; Sh-TSPAN5 1.00±0.04; Rescue 1.38±0.06; DLQ 1.11±0.05; NIYF 1.09±0.04	Scrambled, 44; Sh-TSPAN5, 45; Rescue, 37; DLQ, 45; NIYF, 40 dendrites from 3 different animals per condition
**6**	**B**	cluster/µm^2	Scrambled 0.30±0.02; Sh-TSPAN5 0.18±0.01; Rescue 0.36±0.02; DLQ 0.24±0.01; NIYF 0.25±0.01	Scrambled 19, Sh-TSPAN5 20, Rescue 18, DLQ 27, NIYF 27 neurons
**6**	**B**	average size (µm^2)	Scrambled 0.45±0.03; Sh-TSPAN5 0.33±0.02; Rescue 0.79±0.07; DLQ 0.48±0.02; NIYF 0.33±0.02	Scrambled 19, Sh-TSPAN5 20, Rescue 18, DLQ 27, NIYF 27 neurons
**6**	**D**	global diffusion coefficient (µm^2/s)	Scrambled 0.034±0.002; Sh-TSPAN5 0.065±0.009; Rescue 0.02±0.003; PLM 0.038±0.006	Scrambled 16, Sh-TSPAN5 17, Rescue 16, PLM 12 neurons
**6**	**F**	Stubby (%)	Scrambled non-clustered 33.2±1.6; Scrambled clustered 36.5±2.3; Sh-TSPAN5 non-clustered 46.6±1.6; Sh-TSPAN5 clustered 42.0±2.1	Scrambled non-clustered 10; Scrambled clustered 14; Sh-TSPAN5 non-clustered 16; Sh-TSPAN5 clustered 15 neurons
**6**	**F**	Thin (%)	Scrambled non-clustered 36.6±1.9; Scrambled clustered 28.1±1.5; Sh-TSPAN5 non-clustered 30.1±1.7; Sh-TSPAN5 clustered 29.2±2.2	Scrambled non-clustered 10; Scrambled clustered 14; Sh-TSPAN5 non-clustered 16; Sh-TSPAN5 clustered 15 neurons
**6**	**F**	Mushroom (%)	Scrambled non-clustered 30.2±1.2; Scrambled clustered 35.5±1.9; Sh-TSPAN5 non-clustered 23.3.1±1.4; Sh-TSPAN5 clustered 28.8±1.0	Scrambled non-clustered 10; Scrambled clustered 14; Sh-TSPAN5 non-clustered 16; Sh-TSPAN5 clustered 15 neurons
